# Cytoplasmic mRNA decay by the antiviral nuclease RNase L promotes transcriptional repression

**DOI:** 10.1016/j.celrep.2026.117028

**Published:** 2026-02-25

**Authors:** Xiaowen Mao, Sherzod Tokamov, Felix Pahmeier, Azra Lari, Jinyi Xu, Eva Harris, Britt Glaunsinger

**Affiliations:** 1 Department of Plant and Microbial Biology, UC Berkeley, Berkeley, CA, USA; 2 Division of Infectious Diseases and Vaccinology, School of Public Health, UC Berkeley, Berkeley, CA, USA; 3 Department of Molecular and Cell Biology, UC Berkeley, Berkeley, CA, USA; 4 Howard Hughes Medical Institute, Berkeley, CA, USA; 5 Lead contact

## Abstract

Ribonuclease (RNase) L is an antiviral factor that promiscuously degrades viral and cellular RNA in the cytoplasm. This results in extensive translational reprogramming, altering mRNA processing and export. Here, we reveal that another major consequence of cytoplasmic RNase L activity is the repression of nascent RNA synthesis in the nucleus. This is not associated with altered nuclear RNA stability but instead results from transcriptional repression. For RNA polymerase II, repression is primarily associated with reduced occupancy of serine-2-phosphorylated polymerase in gene bodies, indicating an elongation defect. Prominent among the transcriptionally downregulated loci are immune-related genes, supporting a role for RNase L in tempering innate immune and inflammatory responses. RNase L activation also caused disruption of nucleoli and reduced RNA polymerase I and III transcription. Crosstalk between RNA decay and transcription thereby contributes to the large-scale modulation of gene expression in RNase L-activated cells.

## In brief

RNase L, which promiscuously degrades cytoplasmic RNA, is an important effector of antiviral responses. Mao et al. demonstrate that RNase L activation leads to a repression of transcription, uncovering a significant additional strategy by which RNase L reshapes the cellular gene expression environment.

## Graphical Abstract

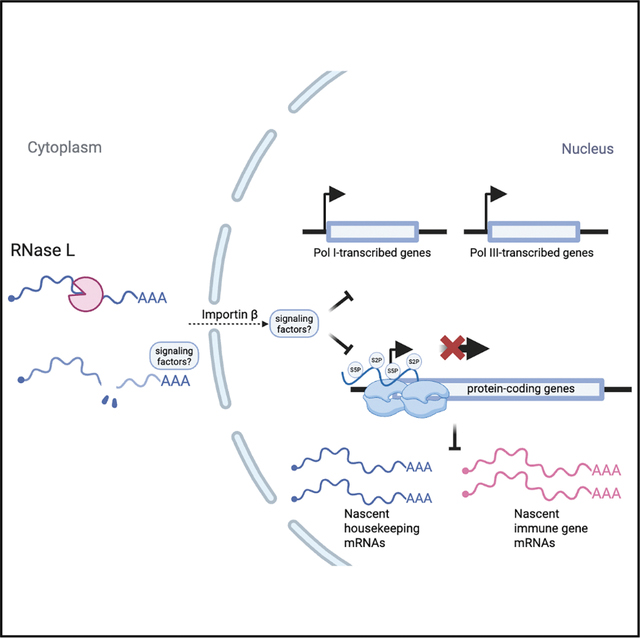

## INTRODUCTION

Ribonuclease (RNase) L is an important player in the innate immune response triggered by double-stranded RNA (dsRNA), a pathogen-associated molecular pattern (PAMP) commonly associated with viral infections. RNase L dimerizes and is activated by 2′-5′-oligo(A), a second messenger produced by oligoadenylate synthetases (OASs) 1–3 upon dsRNA binding. Once activated, RNase L promiscuously cleaves cytoplasmic RNA, including viral RNAs and host rRNA, mRNA, and tRNA, at sites defined by the nucleotide sequence UN ^^^N (^^^ denotes the cleavage site).^1^ RNase L was initially hypothesized to suppress translation through its ability to cleave rRNA and tRNA. However, rRNA and tRNA cleavage occurs after translation remodeling, and ribosomes with RNase L-cleaved rRNAs can still support translation.^2–4^ Thus, widespread cleavage of mRNA most likely causes the prominent decrease in cellular translation.^2,4,5^

In addition to suppressing viral gene expression, RNase L-induced gene expression reprogramming both positively and negatively modulates innate immune responses.^2,4–12^ RNase L promotes virus-induced interferon (IFN)-β production in mice and in cells where RNase L basal expression is low but suppresses IFN-β production in cells with higher basal RNase L and OAS expression, perhaps to promote tissue protection.^10,13^ Additionally, loss-of-function mutations in the OAS-RNase L pathway have been linked to autoimmune diseases, including some cases of multisystem inflammatory syndrome in children following SARS-CoV-2 infection.^9^ These observations suggest that RNase L activity may dampen immune gene expression to prevent overactivation of the innate immune system. However, some mRNAs encoding antiviral factors are at least partially resistant to RNase L and may be preferentially translated due to the liberated translational resources, ensuring the execution of an antiviral response.^2,4,5^ Much remains to be learned about the contexts governing the complex roles of RNase L in immune regulation.

While RNA cleavage is a prominent phenotype associated with RNase L, several recent findings reveal additional mechanisms contributing to its impact on gene expression. These include inhibiting mRNA export,^6^ altering mRNA processing,^7^ activating ribotoxic stress responses,^8,11,14^ and activating RIG-I/MDA5-MAVS pathways through the production of small RNA cleavage fragments.^10,13^ Each of these phenotypes relies on RNase L catalytic activity, suggesting that they occur in response to the widespread RNA cleavage.

Several oncogenic gamma-herpesviruses also engage broad-acting mRNA-targeting nucleases, whose activity, like that of RNase L, can lead to alterations in mRNA processing and export.^15–18^ Notably, viral cleavage of cytoplasmic mRNA also induces large-scale dampening of RNA polymerase II (RNA Pol II) transcription.^19–21^ Similarly, widespread cytoplasmic mRNA decay by the cellular exonuclease polyribonucleotide nucleotidyltransferase 1 (PNPT1), which is activated during early apoptosis, leads to subsequent RNA Pol II transcriptional repression.^22–24^ These findings indicate that there is a positive feedback loop between mRNA synthesis and degradation under conditions of accelerated mRNA decay, which we hypothesized might also occur in response to RNase L activation.

Here, we reveal that activated RNase L represses RNA synthesis. This includes IFN-stimulated genes (ISGs) and other immune genes, suggesting that its impact on RNA biogenesis may help temper inflammatory responses. The reduction of newly synthesized RNA is not due to accelerated RNA decay in the nucleus but instead is triggered indirectly by RNase L-induced RNA degradation in the cytoplasm. This causes a reduction in transcription by RNA Pol I, II, and III, accompanied by alterations in nucleolar and nuclear speckle morphology. The primary alteration to RNA Pol II occupancy is a reduction in the serine-2-phosphorylated (S2P) version in the gene body and near transcription termination sites, suggestive of an elongation defect. The repressive signal involves the transport of proteins into the nucleus, as blocking importin β-mediated nuclear import rescues RNA synthesis in cells with activated RNase L. Thus, crosstalk between the cytoplasm and nucleus promotes broad transcriptional repression upon RNase L activation, which contributes to the reshaping of the gene expression landscape in cells following innate immune stimulation.

## RESULTS

### RNase L activation broadly reduces the abundance of newly synthesized RNA in the nucleus

**Figure 1.**
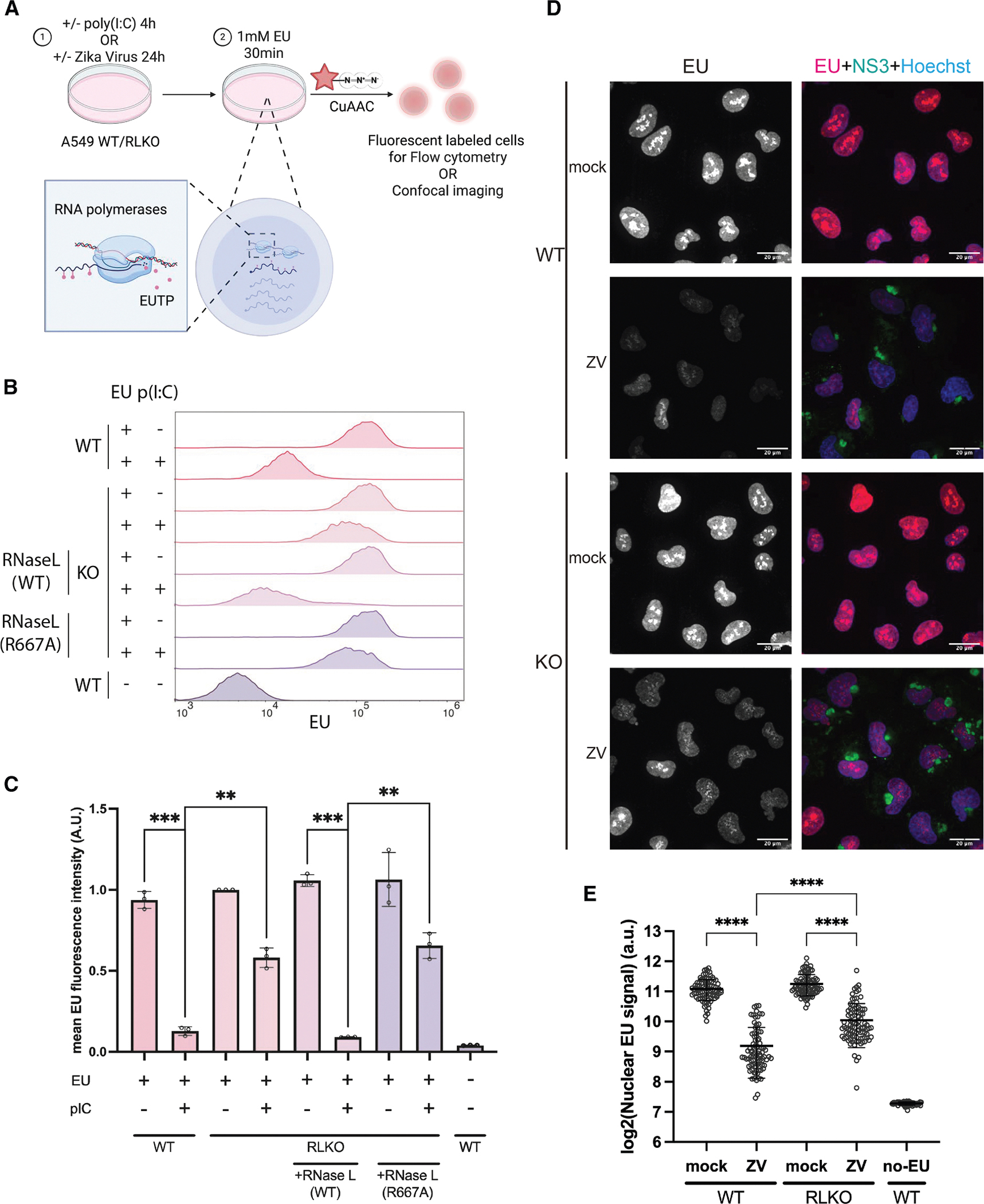
RNase L-mediated mRNA degradation represses nascent RNA accumulation (A) Diagram showing the experimental setup for nascent RNA measurement. (B) Flow cytometry histograms of A549 WT or RLKO cells with the indicated treatment from one representative biological replicate (of 3 independent biological replicates). (C) Quantification of flow cytometry data from 3 independent biological replicates. Each dot represents the median fluorescence intensity of the sample with the indicated treatment from one biological replicate, normalized to the median fluorescence intensity of mock-transfected RLKO cells. Bars represent the mean ± standard deviation (SD). *p* values were calculated using a two-sided Welch’s *t* test. (D) Representative immunofluorescence images (max intensity projection, 3 independent biological replicates) of A549 WT or RLKO cells infected with Zika virus (MOI = 5) at 24 h post-infection (hpi). Cells were stained with a mouse anti-NS3 antibody to indicate infected cells and with Hoechst 33342 to mark nuclei. (E) Quantification of nuclear EU signal in 1 representative replicate of 3 independent biological replicates in (D). Each dot represents the mean nuclear EU fluorescence intensity of one individual nucleus. Bars represent the mean ± SD. The *p* value was calculated using Welch’s *t* test.

Based on observations of positive feedback between RNA decay and synthesis,^19,24^ we sought to determine how RNase L impacts the abundance of newly synthesized RNA in the nucleus. We first designed an assay to quantify the total pool of newly transcribed RNA in response to RNase L activation. We activated RNase L by transfecting human A549 cells with the dsRNA mimic poly(I:C) for 4 h and then added the uridine analog ethynyl uridine (EU) for 30 min to label nascent transcripts. The EU-containing RNA was coupled to a Cy5 fluorophore by click chemistry and then visualized by flow cytometry or imaging (Figure 1A). We confirmed that the labeled RNA remained largely nuclear during the 30-min pulse (Figures S1A and S1B), which is important because, upon export to the cytoplasm, RNA is rapidly degraded by RNase L.

We observed a striking reduction in EU incorporation in poly(I:C)-treated wild-type (WT) A549 cells compared to control mock-treated cells (Figures 1B and 1C). In contrast, poly(I:C) treatment of RNase L-knockout (RLKO) A549 cells resulted in only a modest reduction in EU signal (Figures 1B and 1C), suggesting that RNase L is the primary driver of the loss of newly transcribed RNA in the nucleus. Furthermore, nascent RNA depletion was observed in A549 RLKO cells complemented with WT RNase L but not those complemented with catalytically dead RNase L mutant R667A (Figures 1B and 1C), indicating that this effect required RNase L catalytic activity. This phenotype was detected as early as 2 h after poly(I:C) treatment and grew progressively more pronounced over time (Figure S1C). It was also apparent in WT, but not RLKO, cells treated with a synthetic version of the RNase L-activating second messenger molecule 2–5A (pA(_2–5_A)_4_) (Figure S1D). Poly(I:C) transfection also reduced the EU signal in immortalized murine bone-marrow-derived macrophages (iBMDMs), and this effect was mitigated upon small interfering RNA (siRNA)-mediated depletion of RNase L (Figures S1E–S1G). Although RNase L activation can induce caspase-dependent apoptosis,^12,25^ the irreversible pan-caspase inhibitor z-VAD(Ome)-FMK did not prevent the loss of EU signal (Figure S1H), indicating that repression of nascent RNA does not require caspase activation.

To determine whether these observations occur during viral infection, we performed a 30-min EU pulse during Zika virus infection, which is a potent activator of RNase L.^26–28^ Indeed, Zika virus infection at an MOI of 5 for 24 h resulted in a significant reduction in EU-labeled RNA in WT A549 cells compared to RLKO cells, as visualized by immunofluorescence (Figures 1D, 1E, and S1I). A portion of the Zika virus-induced reduction in nascent RNA was RNase L independent (Figure 1E), which may stem from other effects induced by dsRNA and/or Zika virus capsid protein.^29^ Collectively, these data indicate that RNase L-mediated mRNA degradation represses nascent RNA synthesis in multiple cell types and in response to multiple activating stimuli.

### RNase L reduces both nascent rRNA and pre-mRNA accumulation

**Figure 2.**
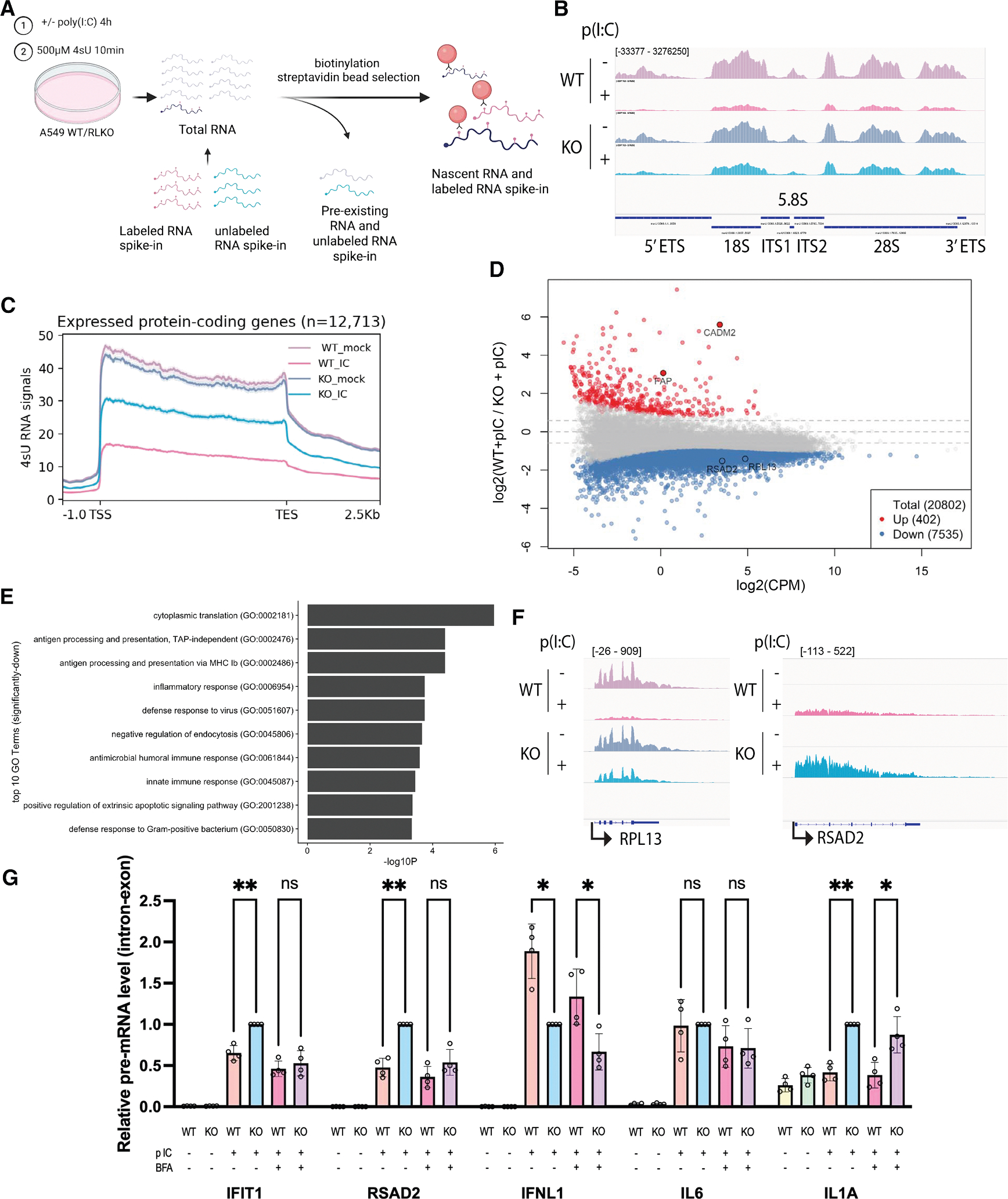
RNase L reduces nascent rRNA and pre-mRNA (A) Diagram summarizing the 4sU-seq experiment. (B) Integrated genome viewer (IGV) tracks showing 4sU-seq coverage at rDNA repeat. Tracks represent the average coverage of 3 independent biological replicates. Coverage depth is shown in brackets in the upper track, with a positive value indicating coverage in the positive strand direction and a negative value indicating coverage in the negative strand direction. (C) Metagene analysis of 4sU-seq signal coverage over expressed protein-coding genes (−1 kb from the transcription start site (TSS) to +2.5 kb from the transcript end site (TES), *n* = 12,713). Lines represent the average coverage of 3 independent biological replicates. (D) Differential expression analysis of 4sU-seq comparing WT cells transfected with poly(I:C) to RLKO cells transfected with poly(I:C). Each gene was plotted with the log_2_(fold change) value on the *y* axis and the log_2_ of the mean expression level, expressed in counts per million (CPM), on the *x* axis. Significantly up (red) and significantly down (blue) are defined as adjusted *p* < 0.05 and log_2_(fold change) > 0.585 or < −0.585, respectively. Dashed lines denote log_2_(fold changes) of −0.585, 0, and 0.585. (E) Gene Ontology (GO) term enrichment analysis of downregulated genes identified in (D). (F) IGV tracks showing 4sU-seq signal coverage on RPL13, an example of ribosomal protein genes, and RSAD2, an example of immune genes. Coverage depth is shown in brackets in the upper track, with a positive value indicating coverage in the positive strand direction and a negative value indicating coverage in the negative strand direction. (G) Pre-mRNA level of immune genes in WT or RLKO cells transfected with poly(I:C), with or without treatment of brefeldin A (BFA). The pre-mRNA level is determined by RT-qPCR using intron-exon-spanning primer pairs, normalized to 18S and poly(I:C)-transfected RLKO cells without BFA. *p < 0.05, **p < 0.01, ***p < 0.001, and ns, not significant. The *p* value was calculated with Welch’s *t* test.

EU is incorporated into transcripts by all three classes of RNA polymerases, with RNA Pol I and II transcripts each accounting for approximately half of the total signal during a short pulse.^30^ Thus, the loss in EU signal caused by RNase L activation is likely due to the reduction of both nascent RNA Pol I and II transcripts. To comprehensively investigate the nascent transcripts affected by RNase L activation, we sequenced the newly transcribed pool of RNA following a 10-min pulse labeling with another uridine analog, 4-thiouridine (4sU). After adding labeled and unlabeled spike-in controls for normalization, the 4sU-incorporated RNA was purified away from the pre-existing pool of RNA by biotinylation of the 4sU and purification via streptavidin beads (Figure 2A). We confirmed >30-fold enrichment of the 4sU-labeled over the unlabeled spike-in RNA in our sequencing data and confirmed 4sU RNA enrichment by RT-qPCR (Figures S2A and S2B).

Initial rRNA filtering revealed that the rRNA reads accounted for no more than 56% of the total reads, which aligns with expectations.^31^ After alignment to the rDNA repeat and spike-in normalization, we observed a decrease in 4sU sequencing (4sU-seq) signal coverage on the entire rDNA repeat in WT cells transfected with poly(I:C), which was less prominent in RLKO cells (Figure 2B). We then aligned the remaining reads to the hg38 reference genome and performed a metagene analysis on protein-coding genes. In agreement with the EU flow cytometry data, we observed a global decrease in nascent mRNA transcripts in poly(I:C)-treated WT cells relative to mock-treated WT cells, as well as in WT cells treated with poly(I:C) compared to RLKO cells treated with poly(I:C) (Figure 2C). We also observed a prominent decrease in several RNA Pol III-transcribed genes (Figure S2C). Differential gene expression analysis of protein-coding and non-coding genes transcribed by all three RNA polymerases revealed a widespread decrease on a gene-by-gene basis, with 36.2% of genes significantly downregulated (Figure 2D). Thus, RNase L plays a major role in repressing the accumulation of nascent rRNA, non-coding RNA, and mRNA.

Fewer genes were significantly upregulated in an RNase L-dependent manner, and manual inspection of these genes revealed that their apparent upregulation is largely explained by one of two situations. The first category contains genes that are differentially expressed in these cells even in the absence of poly(I:C) treatment, indicating that they are the result of minor genetic/epigenetic differences between the parental WT cell line and the clonal RLKO cell line (Figures S2D and S2E). The second category includes genes whose apparent upregulation is instead the result of readthrough transcription from upstream highly expressed genes (Figure S2F). Thus, RNase L activation predominantly causes repression rather than activation of nascent RNA.

Notably, Gene Ontology analysis of the downregulated genes revealed an enrichment of genes involved in cytoplasmic translation and the immune response (Figures 2E and 2F). The translation term is likely due to the reduction in ribosomal protein-encoding mRNAs, which are known targets of RNase L,^32^ suggesting a coordinated response with rRNA transcription inhibition to reduce ribosome biogenesis.^33^ The abundance of immune-related categories suggests that RNase L-induced suppression of nascent RNA contributes to its impact on the immune response. We validated this result by performing RT-qPCR on selected immune transcripts in total RNA samples using intron-exon-spanning primer pairs to measure pre-mRNA. Indeed, ISGs, including IFIT1, RSAD2, OAS1, and OASL, displayed decreased pre-mRNA levels in WT cells compared to RLKO cells stimulated with poly(I:C) (Figures 2G and S2G). In contrast, IFNL1 has previously been shown to undergo intron retention upon RNase L activation, and we observed this expected increase (and a similar trend for ISG15) in WT relative to RLKO cells (Figure 2G).

We next considered the extent to which the decrease in ISG pre-mRNA was due to a reduction in IFN signal amplification versus a cell-autonomous effect, as we observed decreased levels of both nascent IFNB1 and IFNL1. This is also relevant because degradation and/or export inhibition of IFNs and other cytokine cytoplasmic mRNA by RNase L should reduce the level of their encoded secreted proteins, thereby dampening their ability to induce ISG expression in neighboring cells via paracrine signaling. To evaluate this in ISGs, we treated cells concurrently with poly(I:C) and brefeldin A (BFA), which collapses Golgi-endoplasmic reticulum (ER) retrograde transport and inhibits protein secretion.^34^ BFA reduced the difference in nascent mRNA levels between WT and RLKO cells for most of the ISGs we tested, including IFIT1, RSAD2, OAS1, and OASL, confirming the impact of RNase L activity on signal amplification of the IFN pathway (Figures 2G and S2G). However, the difference in IL1A nascent mRNA levels between WT and RLKO cells remained upon BFA treatment (Figure 2G), suggesting that the repression of nascent RNA can independently influence some immune genes in a cell-autonomous manner.

### Reduction of nascent RNA is not due to accelerated nuclear RNA degradation

**Figure 3.**
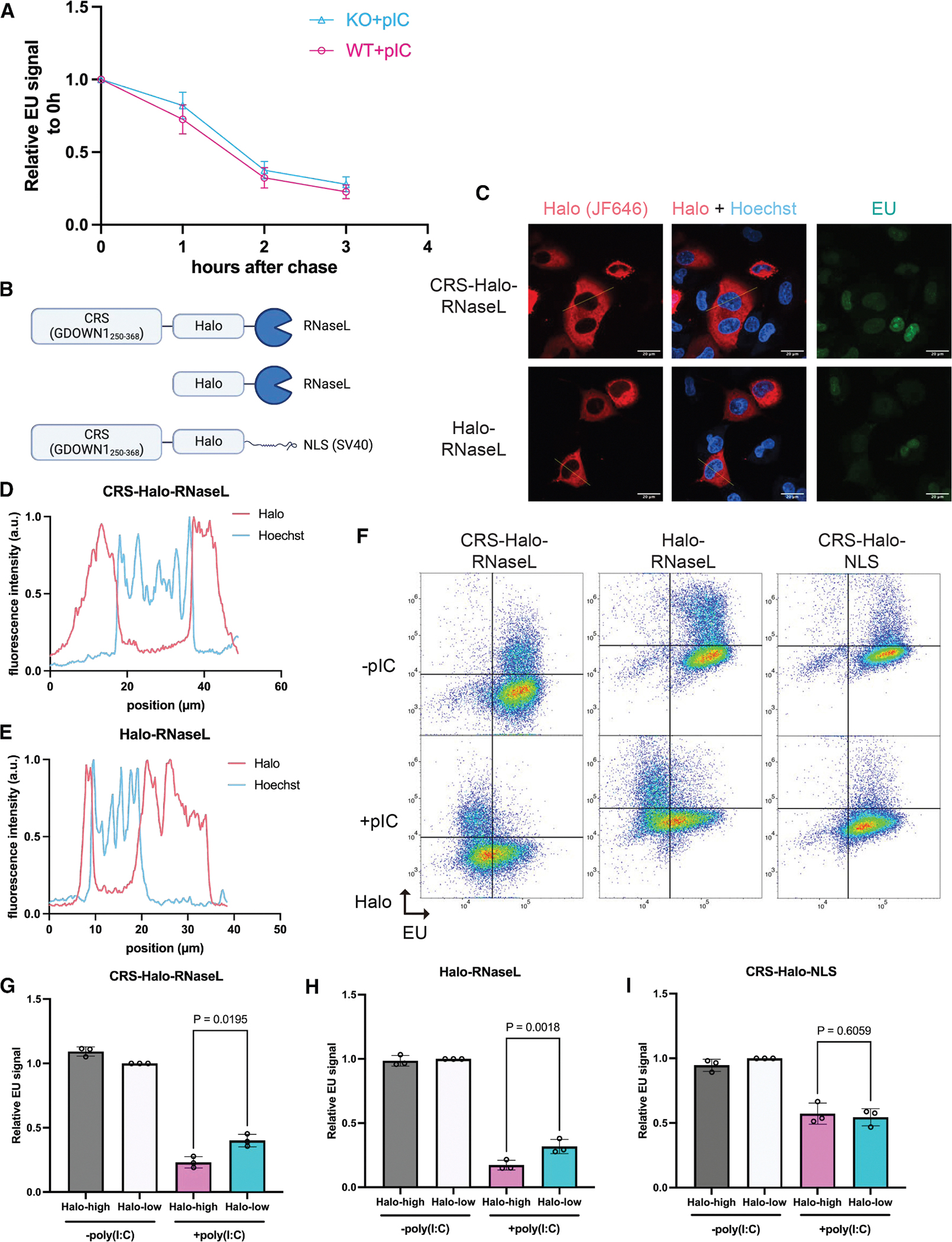
Reduction of nascent RNA is not due to accelerated nuclear RNA degradation by RNase L (A) Quantification of EU signal intensity after uridine chase for durations indicated. Each point represents the average of median EU fluorescence intensity from 3 independent biological replicates, normalized to 0 h chase. Error bars represent mean ± SD. (B) Diagrams of the constructs used. (C) Representative images of the localization of tagged RNase L. The tagged RNase L was visualized by HaloTag labeling with JF646 Halo dye. A single z plane is shown per construct. (D) Line profile of CRS-Halo-RNase L shown in (C). (E) Line profile of Halo-RNase L shown in (C). (F) Flow cytometry scatterplot of A549 RLKO cells nucleofected with the indicated constructs, with or without poly(I:C) transfection. Data are from 1 representative replicate of 3 independent biological replicates. (G–I) Quantification of EU signals in (F). The Halo intensity is gated at locations shown in (F) and separated into 2 populations. Each dot represents the median fluorescence intensity, normalized to the median fluorescence intensity of mock-transfected RLKO cells nucleofected with the constructs indicated. Bars represent the mean ± SD. *p* values were calculated using a ratio paired *t* test.

RNase L is primarily described as a cytoplasmic nuclease^7,35^; however, even a small amount of active RNase L in the nucleus could lead to rapid depletion of pre-mRNA. To directly test whether RNase L activation increases degradation of nascent RNA in the nucleus, we measured nascent RNA turnover using an EU pulse-chase experiment. We pulsed poly(I:C)-transfected cells with EU 1 h after transfection, a time point when EU incorporation is minimally affected by RNase L activation (Figures S1C and S3A). We then chased with excess uridine over a 3-h time course and measured the decay of the labeled RNA. Notably, the rate of RNA decay was similar in A549 WT and A549 RLKO cells, indicating that RNase L does not accelerate nascent RNA decay in the nucleus (Figure 3A). We confirmed that the transfection conditions were sufficient to induce RNase L-specific reduction of nascent RNA (Figures S3A and S3B, see the “pulse last” sample) and that our measurements largely captured nuclear RNA (Figures S3C and S3D).

As an orthogonal way of confirming that the cytoplasmic fraction of RNase L is sufficient to reduce nascent RNA in the nucleus, we complemented the RLKO cells with a Halo-tagged version of RNase L fused to a cytoplasmic retention signal (CRS) (Figure 3B) derived from GDOWN1 (amino acids 250–368).^36^ This CRS promotes cytoplasmic retention by both CRM1-dependent and -independent mechanisms and is strong enough to retain a HaloTag containing an SV40 nuclear localization signal within the cytoplasm (Figure S3E). As expected, both CRS-Halo-RNase L and Halo-RNase L appeared restricted to the cytoplasm (Figures 3C–3E). Furthermore, both constructs reduced EU pulse-labeled nascent RNA levels to a similar extent upon poly(I:C) transfection (Figures 3F–3I), indicating that cytoplasmic RNase L is sufficient to reduce nascent RNA in the nucleus. In contrast, cells overexpressing CRS-Halo-NLS, a negative control, did not exhibit this pattern (Figures 3F and 3I). Taken together, these data suggest that activated RNase L does not induce nuclear RNA decay but rather reduces nascent RNA by affecting transcription.

### RNase L disrupts nucleoli and reduces S5P RNA Pol II levels in the nucleus

**Figure 4.**
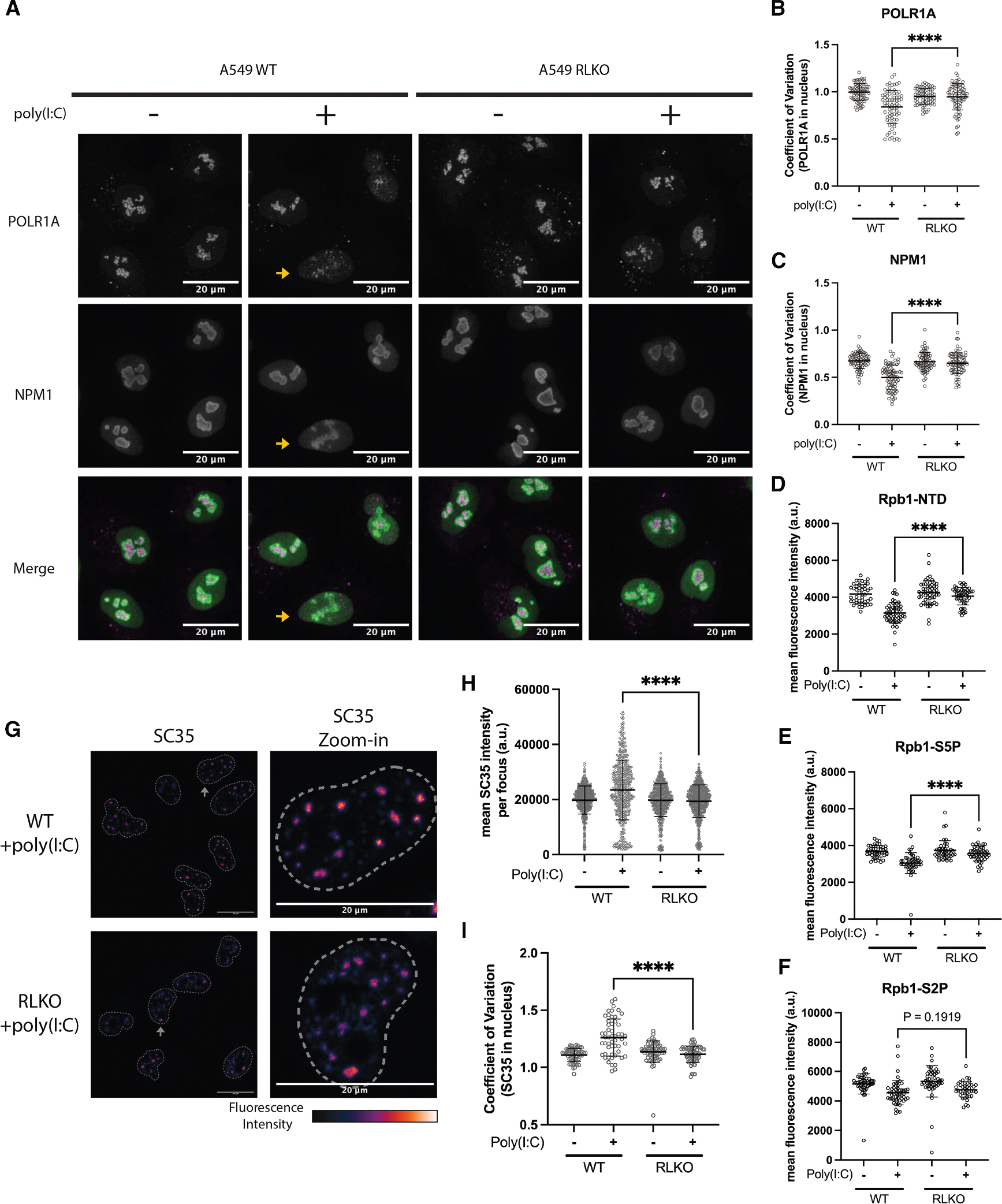
RNase L disperses nucleoli and reduces serine-5-phosphorylated RNA Pol II levels in the nucleus (A) Max-intensity z-projection images showing POLR1A and NPM1 localization in mock- or poly(I:C)-transfected A549 WT or RLKO cells. Yellow arrows point to examples of nuclei showing the dispersal phenotype. (B and C) Quantification of the coefficient of variation (CV) of POLR1A (B) or NPM1 (C) in the nucleus, defined as CV = σ/μ, where σ is the standard deviation and μ is the mean intensity. (D–F) Quantification of mean RNA Pol II (NTD) (D), S5P (E), and S2P (F) staining intensity in poly(I:C)-transfected A549 WT or RLKO cells. (G) Max-intensity z-projection images of SC35 localization in poly(I:C)-transfected A549 WT or RLKO cells. (H and I) Quantification of mean SC35 intensity for each SC35 focus (H) and the coefficient of variation of SC35 in the nucleus (I), using data in (D). In all graphs, each point represents the value calculated for each nucleus. Error bars represent the mean ± SD. ****p < 0.0001 (Welch’s *t* test). The imaging data shown are representative of 3 independent biological replicates.

We first took an imaging-based approach to investigate whether RNase L activity affects the abundance or localization of transcriptional machinery. RNA Pol I resides in the fibrillar component of nucleoli, which is surrounded by the nucleophosmin 1 (NPM1)-containing granular component. Using antibodies against the large POLR1A subunit of RNA Pol I and NPM1, we confirmed that POLR1A formed large aggregates surrounded by a ring-like pattern of NPM1 in mock-treated WT and RLKO cells (Figure 4A; Videos S1 and S2). While the overall abundance of POLR1A and NPM1 did not significantly change upon poly(I:C) treatment (Figures S4A and S4B), POLR1A aggregates and NPM1 rings became more dispersed in many of the WT cells but less so in the RLKO cells (Figure 4A, yellow arrow denotes a WT cell exemplifying this phenotype; Videos S3 and S4). We quantified this dispersion by calculating the coefficient of variation (defined as CV = σ/μ, where σ is the standard deviation and μ is the mean intensity) of POLR1A and NPM1 signals in the nucleus, with a lower CV indicating a more even (i.e., dispersed) distribution of signals. This confirmed a significantly reduced mean CV for both POLR1A and NPM1 in poly(I:C)-treated WT cells compared to RLKO cells (Figures 4B and 4C). Thus, consistent with the reduced RNA Pol I-mediated rRNA transcription observed by 4sU-seq, RNase L activation leads to nucleolar disruption.

We next monitored the large RPB1 subunit of RNA Pol II, which undergoes progressive phosphorylation within the heptad repeats in its C-terminal domain (CTD). We used antibodies recognizing either the RPB1 N terminus (N-terminal domain [NTD], all phosphorylation states), the CTD S5P version associated with transcription initiation, and the CTD S2P version associated with elongation. Poly(I:C) treatment caused a modest but significant decrease in the abundance of total (NTD) and S5P RNA Pol II in WT but not RLKO cells (Figures 4D, 4E, and S4C). We further validated the reduction of S5P RNA Pol II by using an independent antibody (3E8) (Figure S4D). We did not observe consistent changes to total or phosphorylated RNA Pol II localization (Figure S4C) or to the abundance of S2P RNA Pol II upon poly(I:C) treatment (Figure 4F).

Finally, we analyzed nuclear speckles, which are hubs for RNA Pol II co-transcriptional RNA processing and undergo morphological changes in transcriptionally inhibited cells.^37^ Although nuclear speckle size was unchanged (Figure S4E), there was an increase in SC35 intensity and CV in speckles in poly(I:C)-treated WT, but not RLKO, cells (Figures 4G–4I and S4F), as well as a more rounded speckle morphology (Figure S4G). Thus, RNase L activation is associated with a modest reduction in the abundance of unphosphorylated and S5P RNA Pol II, as well as morphological changes to nuclear speckles and nucleoli.

### RNase L represses transcription via the reduction of RNA Pol II occupancy in the gene body

**Figure 5.**
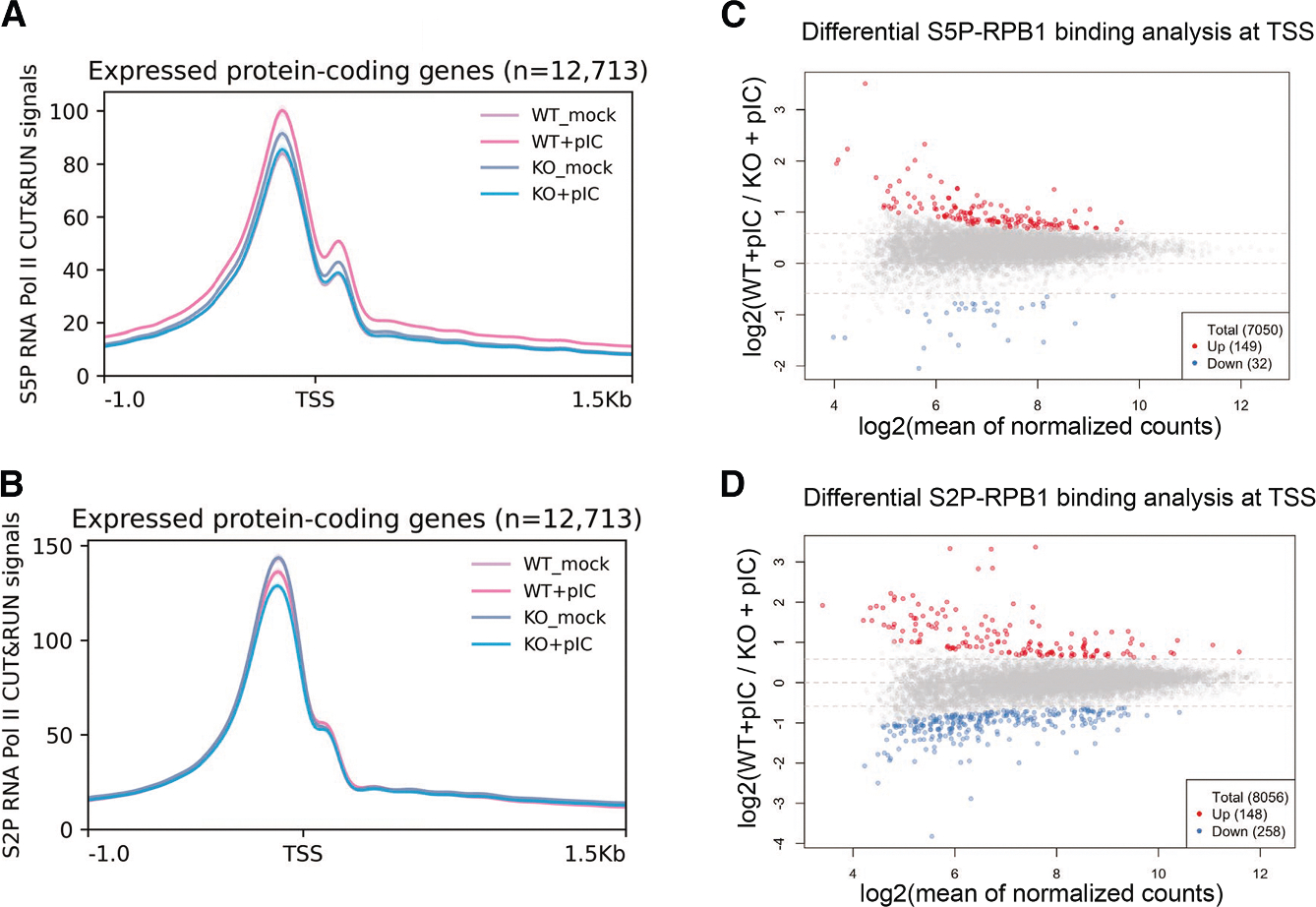
RNase L activation does not broadly inhibit RNA Pol II occupancy at TSSs (A and B) Metagene analysis of S5P (A) and S2P (B) RNA Pol II occupancy at −1 to +1.5 kb around TSS on expressed protein-coding genes in WT or RLKO cells, either mock transfected or transfected with poly(I:C). Lines represent the average coverage of 3 independent biological replicates. (C and D) Differential binding analysis of S5P (C) and S2P (D) RNA Pol II CUT&RUN-seq signal at the TSS (−500 to +250 bp) comparing WT cells transfected with poly(I:C) to RLKO cells transfected with poly(I:C). Significantly up (red) and significantly down (blue) are defined as adjusted *p* < 0.05 and log_2_(fold change) > 0.585 or < −0.585, respectively. Dashed lines denote log_2_(fold changes) of −0.585, 0, and 0.585.

To directly measure how RNase L activation influences RNA Pol II occupancy genome wide, we performed cleavage under targets and release using nuclease followed by sequencing (CUT&RUN-seq) using antibodies to the RNA Pol II S5P CTD (4H8) and RNA Pol II S2P CTD (E1Z3G) and applied yeast spike-in DNA normalization to account for potential global changes in occupancy. S5P and S2P RNA Pol II occupancy was not globally changed at the transcription start site (TSS) in poly(I:C)-treated WT compared to RLKO cells or untreated cells (Figures 5A and 5B). Analyzing subnucleosomal fragments (<120 bp) and nucleosomal fragments (>120 bp) separately yielded similar results (Figures S5A and S5B), as did a differential binding analysis using diffBind to measure RNA Pol II occupancy on a gene-by-gene basis (Figures 5C and 5D).

**Figure 6.**
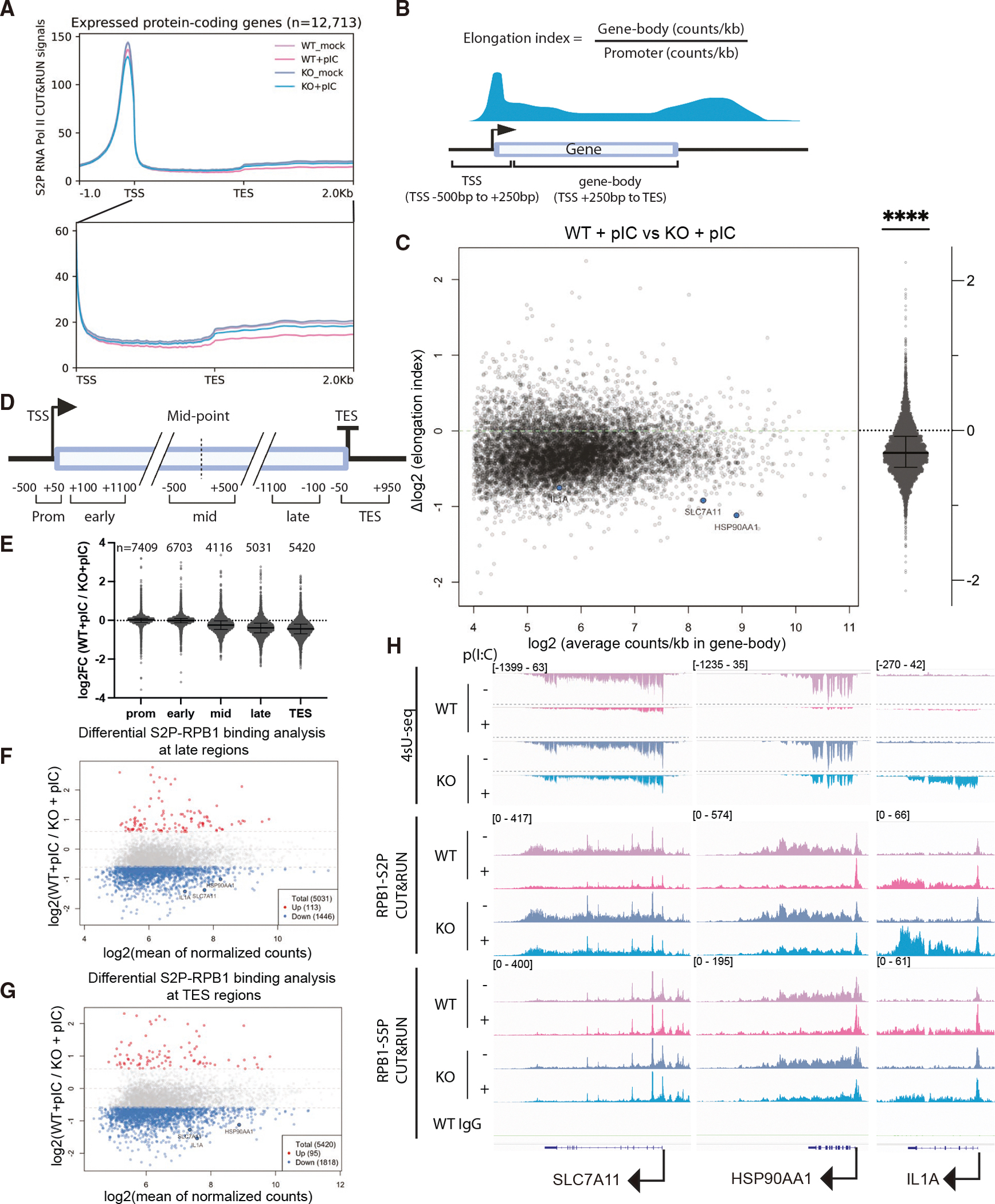
RNase L activation reduces S2P RNA Pol II occupancy in the gene body (A) Metagene analysis of S2P RNA Pol II occupancy on expressed protein-coding genes in WT or RLKO cells, either mock transfected or transfected with poly(I:C). The bottom image represents a zoomed-in view of coverage from the TSS to 2 kb after the TES. Lines represent the average coverage of 3 independent biological replicates. (B) Diagram depicting the calculation of the elongation index and the range of the TSS window and the gene-body window. (C) Scatterplot showing the changes in the elongation index in relation to the read density in the gene body. The median and interquartile range of the changes in elongation index are shown to the right. ****p < 0.0001 using a one-sample *t* test, comparing to 0. (D) Diagram depicting the range of promoter (Prom), early-elongation (early), mid-elongation (mid), late-elongation (late), and TES windows. (E) Summary of log_2_(fold changes) comparing WT to RLKO cells, both transfected with poly(I:C), at windows defined in (D). Each dot represents the log_2_(fold change) of S2P binding at the corresponding window within a gene, calculated by diffBind. Only genes longer than 3.2 kb were included to minimize overlap. *n* represents quantifiable loci retained by diffBind after filtering. Error bars represent the median and interquartile range. (F and G) Zoom-in view of the differential binding analysis in (E) of S2P RNA Pol II CUT&RUN signal at the late elongation windows (−1,100 to −100 bp from TES) (F) or TES windows (−50 to +950 bp from TES) (G). Significantly up (red) and significantly down (blue) are defined as adjusted *p* < 0.05 and log_2_(fold change) > 0.585 or < −0.585, respectively. Dashed lines denote log_2_(fold changes) of −0.585, 0, and 0.585. (H) IGV tracks showing RNA Pol II 4sU-seq, S2P CUT&RUN, and S5P CUT&RUN signal coverage on the housekeeping genes SLC7A11 and HSP90AA1 and the immune gene IL1A. All tracks represent the average coverage of 3 independent biological replicates. Coverage depth is shown in brackets in the upper track. For 4sU-seq tracks, a positive value indicates coverage in the positive strand direction and a negative value indicates coverage in the negative strand direction.

In contrast to the TSS occupancy data, when we zoomed in on the gene body and transcript end site (TES) regions, we observed a reduction in S2P RNA Pol II occupancy (Figure 6A). To quantify this reduction, we first calculated an elongation index, defined as the density of reads in the gene body (+250 bp from the TSS to the TES) divided by the density of reads at the TSS (−500 to +250 bp from the TSS) (Figure 6B), with a lower elongation index suggestive of a defect of S2P RNA Pol II traveling into the gene body. We observed an overall reduced elongation index in poly(I:C)-treated WT cells compared to poly(I:C)-treated RLKO cells or mock-treated cells (Figures 6C, S6A, and S6B). This observation was further supported by a differential binding analysis across windows spanning different gene regions, which showed a progressive reduction of S2P RNA Pol II binding midway through the gene body to the TES (Figures 6D and 6E). Approximately 30% of expressed genes show reduced S2P RNA Pol II binding at the late-elongation window and the TES window (Figures 6F and 6G), and these also have a more pronounced reduction in 4sU RNA level (Figures S6C and S6D). Example traces are shown in Figure 6H, demonstrating reduced S2P occupancy within the gene body but no reduced S2P or S5P occupancy at the TSS of poly(I:C)-treated WT relative to RLKO cells. While it is possible that the loss of S2P RNA Pol II signal is due to reduced phosphorylation or enhanced dephosphorylation, such a reduced S2P mark is also indicative of affected RNA Pol II elongation, given the pivotal roles of the S2P mark in regulating RNA Pol II elongation.^38–40^ Thus, RNase L activation negatively impacts RNA Pol II transcription primarily during the elongation phase.

### Importin β-mediated nuclear import links cytoplasmic RNA decay to transcription

**Figure 7.**
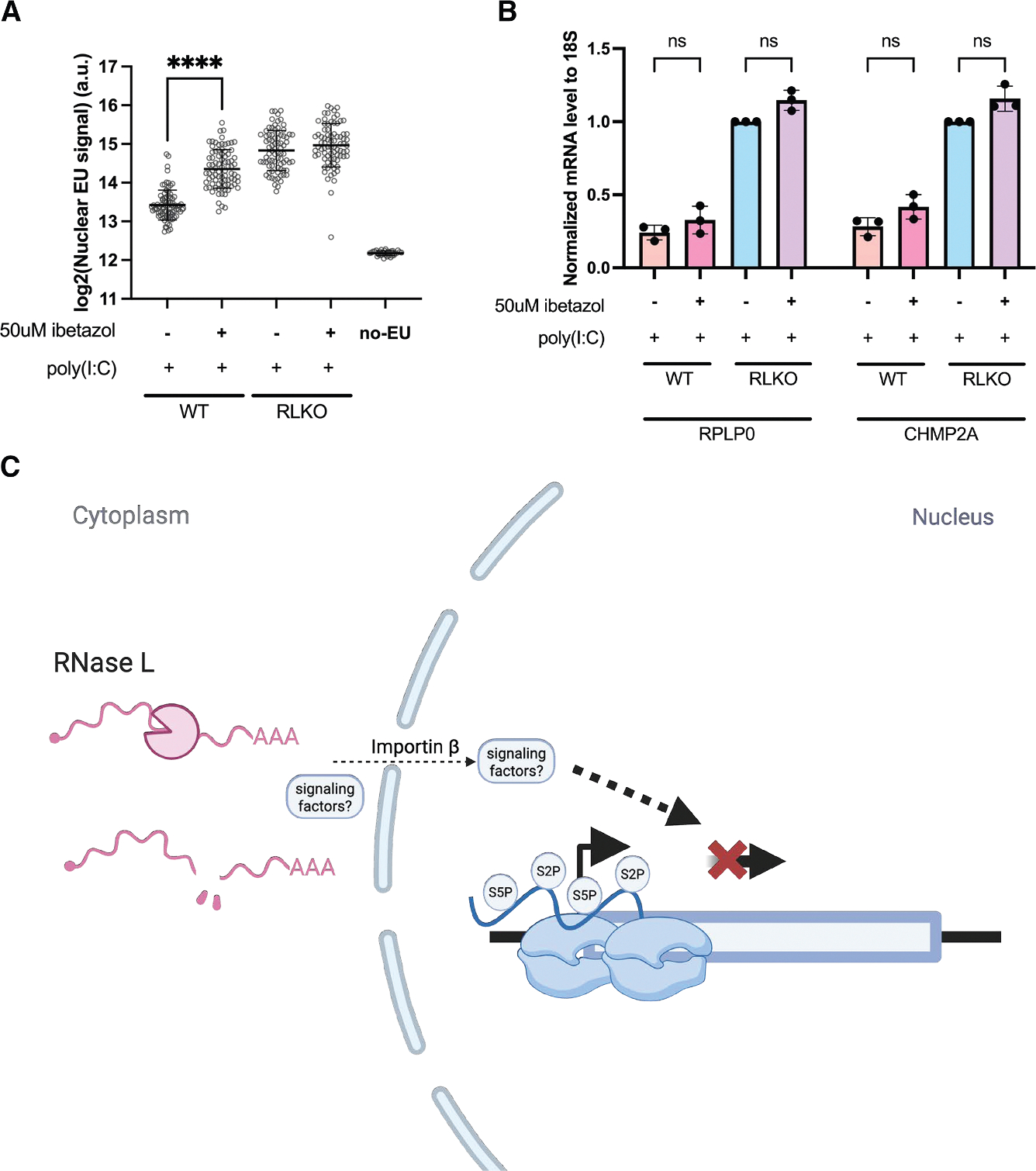
Inhibition of importin-β dampens transcription repression by RNase L (A) Quantification of nuclear EU signal in WT or RLKO cells with indicated treatments. The data represent 1 representative replicate of 4 independent biological replicates. Each dot represents the mean nuclear EU fluorescence intensity of one individual nucleus. Bars represent the mean ± SD. ****p < 0.0001. The *p* value was calculated using Welch’s *t* test. (B) RT-qPCR measuring the indicated housekeeping mRNA after poly(I:C) transfection in WT or RLKO cells, with or without ibetazol. ns, not significant (*p* > 0.05 was calculated with two-sided Welch’s *t* test). (C) Diagram depicting a model of RNase L-dependent transcription repression.

Finally, we sought to determine the nature of the signal linking RNase L-induced cytoplasmic RNA decay with transcriptional repression in the nucleus. Accelerated cytoplasmic RNA decay is known to cause differential trafficking of RNA-binding proteins (RBPs) from the cytoplasm to the nucleus.^7,20^ To determine whether protein nuclear import plays a role in transcriptional repression by RNase L, we treated cells with ibetazol, a specific inhibitor of importin β-mediated nuclear import,^41^ prior to measuring EU incorporation in our pulse labeling assay. Indeed, ibetazol caused a significant increase in EU incorporation in poly(I:C)-treated WT cells but did not alter EU incorporation in RLKO cells (Figure 7A). Importantly, RNase L retained its mRNA degradation activity in ibetazol-treated cells, as measured by RT-qPCR for RPLP0 and CHMP2A (Figure 7B). Thus, nuclear import of proteins, potentially those released during cytoplasmic RNA decay by RNase L, is required to induce subsequent transcriptional repression.

## DISCUSSION

RNase L-induced cytoplasmic mRNA degradation extensively remodels the cellular gene expression landscape, influencing how cells respond to viral infection. Although originally thought to mainly inhibit translation, it is now appreciated that RNase L triggers cascading impacts on upstream stages of gene expression, including mRNA processing and export.^6,7^ Here, we demonstrate through EU pulse labeling, nascent mRNA sequencing, and RNA Pol II occupancy experiments that cytoplasmic RNase L activity also profoundly impacts RNA synthesis by repressing transcription, including at immune genes. RNase L-induced transcriptional repression coincides with nucleolar disruption, as well as changes in nuclear speckle morphology, similar to those previously reported in transcriptionally inhibited cells.^37^ Decay-to-transcription signaling involves protein shuttling between the two compartments, as inhibition of importin β breaks this connection and restores RNA synthesis (Figure 7C). The combination of these disruptions to the whole cellular gene expression pathway, spanning RNA synthesis through to translation and decay, is likely what renders RNase L such a potent modulator of gene regulation.

We hypothesize that RNase L affects nascent mRNA primarily by impairing transcription elongation, as the most prominent RNA Pol II phenotype was reduced S2P RNA Pol II occupancy at the 3′ end of genes. However, it is possible that other stages of RNA biogenesis may also be impacted, especially given our imaging data showing a modest reduction in RNA Pol II levels within the nucleoplasm. The fact that we observed reduced S5P RNA Pol II levels by immunofluorescence but not by CUT&RUN suggests that a proportion of free or unstably bound S5P RNA Pol II may undergo degradation. This could, for example, be mediated by the ARMC5 E3 ligase if the RNA Pol II is defective.^42–44^ It is possible that, over time, a gradual reduction in total RNA Pol II could lead to additional effects at earlier stages of transcription or that reduced S5P RNA Pol II selectively, rather than globally, impacts early-stage transcription in RNase L-activated cells.

Our observation that RNase L activation leads to changes to RNA Pol I, NPM1, and SC35 distribution is consistent with previous reports that inhibition of RNA Pol I and II transcription can cause morphological changes to their respective transcription and processing sites.^45–47^ Notably, the dispersion of RNA Pol I and NPM1 was similarly reported in cells treated with the cyclin-dependent kinase (CDK) inhibitor flavopiridol and was potentially due to inhibition of the phosphorylation of RNA Pol I co-activator Treacle.^45^ Nuclear speckle-associated proteins play roles in co-transcriptional RNA processing. Similar to RNA Pol II inhibition by chemical treatment, RNase L activation also altered nuclear speckle morphology, suggesting a link between transcription repression and other co-transcriptional defects, including intron retention^7^ and reduced export^6,46^ in RNase L-activated cells.

The fact that RNase L promotes transcriptional repression from the cytoplasm indicates that its mechanism of action is indirect and requires one or more signals to be conveyed between the compartments. We hypothesize that this involves the relocalization of RBPs from the cytoplasm to the nucleus, given that blocking protein nuclear import significantly increases RNA synthesis in RNase L-activated cells. More than 20 RBPs shuttle into the nucleus upon their release from mRNA undergoing degradation by herpesviral nucleases,^20^ and several similarly relocalize in RNase L-activated cells.^7^ Nuclear accumulation of these RBPs alters co-transcriptional RNA processing, including promoting mRNA hyperadenylation and nuclear poly(A) RNA accumulation.^6,7,15,48^ It is also associated with transcriptional repression by herpesviral nucleases.^19–21^ Thus, one possibility is that the accumulation of these RBPs in the nucleus disrupts co-transcriptional processing or the function of transcriptional regulators, leading to the observed elongation defects.

Other signals, in addition to protein redistribution, could also contribute to transcriptional repression by RNase L. For example, it could be propagated by stress signals conveyed by the global disruption of protein translation, e.g., as a consequence of ZAKα-dependent ribotoxic stress responses or other ribosome quality control pathways induced by RNase L.^8,11^ In support of this hypothesis, nascent protein ubiquitinylation in response to disrupted translation during heat shock contributes to transcriptional repression via a mechanism involving p38 signaling.^49^ Additionally, RNase L-induced translation arrest could result in a loss of proteins involved in global transcription regulation, particularly those with shorter half-lives. Consistent with this possibility, translation inhibition by cycloheximide in mouse embryonic stem cells can lead to depletion of euchromatin modifiers such as Chd1 and reduced euchromatin marks, reduced RNA Pol II occupancy, and less nascent RNA.^50^ However, our observation that nascent RNA synthesis was reduced as early as 2 h post-poly(I:C) transfection indicates that only highly labile proteins should be affected. Finally, it is possible that RNA fragments generated by RNase L cleavage serve as signaling molecules in the cytoplasm or upon transit into the nucleus.^51^

A key outcome of transcriptional repression is the modulation of immune gene transcription. We find that although ISGs are robustly induced by poly(I:C) transfection independently of RNase L, RNase L activity tempers their induction. Many ISGs are downregulated due to reduced IFN signal amplification, which is relevant considering that RNase L activation is not uniform in cultures of cells exposed to dsRNA, and thus decreased gene expression by RNase L should influence innate immune signaling in neighboring cells.^4,6,52^ However, there are immune genes, such as IL1A, whose transcription is directly repressed by cytoplasmic RNA decay, indicating that RNase L-induced transcriptional repression can have both cell-autonomous and paracrine effects. Given the clinical implications of OAS and RNase L mutations in autoimmune diseases, including MIS-C,^1,9^ elucidating how the RNA-decay-driven transcription repression shapes the immune landscape—in coordination with other branches of RNase L-driven pathways—should remain a focus of future studies.

### Limitations of the study

While our data support a defect in RNA Pol II elongation in response to RNase L activation, we cannot rule out additional possible defects in other stages of transcription, including changes in CTD phosphorylation, initiation, and pause release. We also do not know whether the transcriptional defects observed for RNA Pol I and III occur via a similar or distinct mechanism from that observed for RNA Pol II. Finally, while we hypothesize that RBPs that are released from degraded RNA in the cytoplasm mediate decay-to-transcription signaling during RNase L activation, an important future goal is to determine which specific proteins are required for transcriptional repression.

## RESOURCE AVAILABILITY

### Lead contact

Requests for further information, resources, and reagents should be directed to and will be fulfilled by the lead contact, Britt Glaunsinger (glaunsinger@berkeley.edu).

### Materials availability

Plasmids generated in this study have been deposited to Addgene (see key resources table for accession numbers).

### Data and code availability

4sU-seq, S5P RNA Pol II CUT&RUN-seq, and S2P RNA Pol II CUT&RUN-seq datasets were deposited in NCBI Gene Expression Omnibus under the accession numbers GEO: GSE298174, GSE298175, and GSE313712, respectively. No original code was reported. Further information required for the reanalysis of data reported here will be available upon request.

## STAR★METHODS

### Experimental model and study participant details

A549 WT, RLKO, RLKO-RL and RLKO-CM cell lines^2^ were generously provided by James Burke (University of Florida Scripps) and Roy Parker (University of Colorado, Boulder). Immortalized murine bone marrow macrophages^55^ were generously provided by Susan Carpenter (UC Santa Cruz). All cell lines were maintained in DMEM supplemented with 10% FBS and tested for mycoplasma routinely using Universal Mycoplasma Detection Kit (ATCC, cat # 50189644FP).

## METHOD DETAILS

### Plasmids

All plasmids generated in this study were sequence verified by whole plasmid sequencing and have been deposited on Addgene, with accession numbers indicated in the key resources table. All PCR reactions were performed using Q5 2X master mix (NEB, cat #M0494S). Sequences encoding the CRS tag, HALO tag, and RNase L with appropriate overhangs were PCR amplified. pLJM1-Halo-RNaseL was constructed by directly cloning the HALO and RNase L PCR fragments into linearized pLJM1-puro^67^ using InFusion (Takara Bio, Cat # 639650). pLJM1-CRS-Halo-RNaseL was constructed by first assembling CRS, HALO and RNase L PCR fragments into a single fragment by overlap extension PCR, followed by cloning into linearized pLJM1-puro using InFusion. pLJM1-CRS-Halo-NLS was generated from pLJM1-CRS-Halo-RNaseL using a site-directed mutagenesis protocol described previously^68^ to replace RNase L fragment with an SV40 NLS (PKKKRKV). pLJM1-Halo-NLS was generated from pLJM1-Halo-RNaseL using the same mutagenesis method as above.

### EU pulse-chase for flow cytometry

Cells were were mock-transfected, transfected with 0.76 μg/ml poly(I:C) HMW (InVivoGen, cat # tlrl-pic), or 10 μM 2–5A (pA(_2–5_A)_4_, custom synthesized by IDT) using Lipofectamine RNAiMAX (Thermo Fisher, cat # 13778–150) as indicated, then the media was replaced with 1mM EU (Click Chemistry Tools cat #CCT-1261–500 or Lumiprobe cat # 2439–500mg) in DMEM with 10% FBS. The cells were incubated at 37°C for 30 min. For flow cytometry samples, cells were washed once with 1 mL PBS, harvested, and fixed with 1% PFA. The cells were then permeabilized by resuspending in 100 μl 0.05% w/v saponin in PBS. To visualize EU incorporation, 500 μl click reaction mix (4 mM CuSO_4_, 100 mM sodium ascorbate, 2 μM sulfo-cyanine5-azide (Lumiprobe cat# A3330) in TBS, 50 mM Tris-HCl, pH 8.0, 150 mM NaCl) was added to the cell suspension and the samples were incubated at room temperature for 30 min, protected from light. The samples were then washed twice with 1 mL 0.05% w/v saponin in PBS, once with 1 mL PBS, then resuspended in PBS before flow cytometry analysis. The samples were then analyzed on an BD Accuri C6 Plus flow cytometer.

For EU pulse-chase experiments, WT cells were first color-labeled with 1 μM CFDA-SE (Cayman Chemical, cat # 14456) in PBS for 10 min at room temperature, quenched with equal volume of DMEM with 10% FBS, then washed with DMEM with 10% FBS 3 times. The labeled WT cells were resuspended in DMEM with 10% FBS, pooled with unlabeled RLKO cells at a 1:1 ratio and were seeded in 6-well plates the day before experiment. Cells were transfected with 0.76 μg/ml poly(I:C) HMW (InVivoGen, cat # tlrl-pic) using Lipofectamine RNAiMAX (Thermo Fisher, cat # 13778–150) for 1 h and pulsed with 1 mM EU in DMEM with 10% FBS for 30 min at 37°C. The EU-containing media was removed, and cells were washed with PBS once and immediately chased with 10 mM uridine in DMEM with 10% FBS. The samples were harvested at the indicated time-points. An additional well without transfection and EU pulse labeling was harvested at the same time as the 0-h chase samples, serving as a no-EU control. The “Pulse last” sample, which served as a control for transfection efficiency, was transfected with 0.76 μg/ml poly(I:C) for 1 h, incubated DMEM with 10% FBS without EU for 30 min at 37°C, washed with PBS once and replaced the supernatant with DMEM with 10% FBS and incubated for 1.5 h. Then the cells were pulsed with 1mM EU in DMEM with 10% FBS for 30 min at 37°C and harvested at the same time as the 2-h chase samples.

To characterize EU incorporation in cells overexpressing tagged RNase L, A549 RLKO cells were first nucleofected with the indicated construct using Neon transfection system (Invitrogen) according to the manufacturer’s recommended settings for A549 cells (1230V, 30 ms, 2 pulses), using 5 μg DNA per 750,000 cells in 100 μL buffer R. The nucleofected cells were cultured in 6-well plates overnight. The cells were then incubated with 20 nM Halo ligand Janelia Fluor 646 (Promega, cat # GA1121) diluted in 1mL DMEM +10%FBS per well 30 min prior to poly(I:C) transfection, then transfected with 0.76 μg/ml poly(I:C) for 4 h without removing the Halo dye and pulsed with 1 mM EU for 30 min same as above. The samples were processed as above, with the exception that 5 μM Alexa Fluor 488 Azide (Thermo Fisher, cat #A10266) was used.

To characterize EU incorporation in murine iBMDM cells depleted of RNase L, cells were maintained in DMEM with 10% FBS and grown to 90% confluence. Cells were removed and washed once with Dulbecco’s phosphate-buffered saline (DPBS) (Gibco). Nucleofections were done using the Neon Transfection System (Thermo Fisher). 2 × 10^6^ cells were resuspended in 100 μL of buffer R, to which control non-targeting or RNase L targeting pools of siRNA (ON-TARGETplus SMARTpool siRNA – Horizon Discovery) were added to a final concentration of 200 nM. This was loaded into a Neon 100-μL pipette tip and Neon tube with 3 mL of buffer E2 with electroporation parameters set to 1,680 V, 20 ms, 1 pulse. Following electroporation, cells were plated in 10 mL of DMEM with 10% FBS in 10 cm TC-treated plates and were incubated at 37°C for 24 h. Cells were then transfected with 0.76 μg/ml poly(I:C) pulsed with 1mM EU for 30 min same as above. The samples were processed as above.

### Immunofluorescence

For Zika infection in Figures 1D and 1E and S1G, A549 WT or RLKO cells were seeded in glass-bottomed 24 well-plates at a density of 100,000 cells per well the day before the experiment, and after media change, were infected with Zika virus (Nica 2–16, GenBank accession KX421194.1) at an MOI = 5 for 24 h. Cells were treated with 1 mM EU for 30 min before being washed once with 1 mL of PBS, then fixed with 4% PFA for 15 min. The cells were then permeabilized with 0.5% Triton X-100 in PBS for 15 min and washed 3 times with PBS. Samples were then incubated with 500 μL of click reaction mix at room temperature for 30 min, followed by two washes with PBS and storage in PBS. Samples were then blocked with 1% BSA in PBS with 0.02% Tween 20 for 1 h and incubated with mouse anti-NS3 antibody (Clone E1D8) diluted 1:100 in the blocking solution.^53^ The samples were then washed three times with PBS + 0.02% Tween 20, incubated with goat anti-mouse IgG AlexaFluor Plus 488 (Invitrogen, cat #A32723) diluted 1:1000 in blocking solution, and washed three times with PBS +0.02% Tween 20. The nuclei were stained with 8 μM Hoechst 33342 in PBS for 30 min, followed by 2 PBS washes.

For RNase L localization studies, A549 RLKO cells were nucleofected as in the “EU pulse chase for flow cytometry” section and 340,000 cells were seeded per well in a glass-bottomed 24-well plate and incubated overnight. Cells were first labeled with 20 nM Halo ligand Janelia Fluor 646 for 30 min and transfected with poly(I:C) at a concentration of 0.76 μg/ml for 4 h without removing the Halo ligand. The cells were then pulse labeled with EU and fixed, permeabilized, labeled with click reaction and Hoechst 33342 as above.

For characterizing changes in POLR1A, NPM1, RPB1 and nuclear speckle morphology, A549 WT or RLKO cells were seeded in glass-bottomed 24 well-plates at a density of 100,000 cells per well the day before experiment and transfected with either lipofectamine RNAiMAX alone or with poly(I:C) at a concentration of 0.76 μg/ml for 4 (optionally pulsed with 1 mM EU for 30 min) to 4.5 h. Samples fixed and permeabilized and optionally labeled with click reaction as above, then were blocked with 0.3%–0.5% BSA in PBS with 0.3–0.5% Triton X-100 for 0.5–1 h. Then samples were stained with primary antibodies against POLR1A (clone D6S6S, Cell Signaling, #24799, 1:500), NPM1 (Abcam, ab10530–100, 1:500), Rpb1 NTD (clone D8L4Y, Cell Signaling Technology, 1:500), Rpb1 S5P (ab5131, Abcam, 1:500; or 3E8, Active Motif, RRID:AB_2687451), Rpb1 S2P (ab5095, Abcam, 1:500) and SC35 (ab11826, Abcam, 1:500) diluted in blocking solution overnight at 4°C, goat anti-rabbit IgG AlexaFluor Plus 488 (Invitrogen, cat #A32723) alone or goat anti-mouse IgG Alexa Fluor 546 (Invitrogen, cat # A11035), donkey anti-rat IgG AlexaFluor Plus 488 (Invitrogen, cat # A48269), and goat anti-rabbit IgG Alexa Fluor 647 (Invitrogen, cat # A27040) diluted 1:1000 in blocking solution as secondaries, and 8–10 μM Hoechst 33342.

For Ibetazol treatment, A549 WT or RLKO cells were seeded in glass-bottomed 24 plates at a density of 100,000 cells per well the day before the experiment and transfected with poly(I:C) at a concentration of 0.76 μg/ml for 3 h in the presence of DMSO or 50 μM Ibetazol. Cells were then pulse labeled with EU and fixed, permeabilized, labeled with click reaction.

All samples were imaged on a Nikon Eclipse Ti spinning disk confocal microscope or a Zeiss LSM 880 laser scanning microscope.

### Image processing

All confocal image processing was performed using FIJI,^65^ either manually or with custom macros. Line profile measurements were performed using the plot profile function, and the data were exported to Prism 10 for normalization and visualization. For nuclear signal quantification in Figures 1D and 1E and 4D–4F, the nuclear regions of interest (ROIs) were generated from the Hoechst channel in the image z stack by Gaussian blur, thresholding with the Otsu method, and 3D segmentation using the 3D Manager. To fill holes within ROIs and exclude objects on edges, the 3D segmentation map was converted to binary, followed by “3D fill holes” and “3D exclude edges”. The processed binary map was 3D segmented again using the 3D Manager to generate the final ROIs. The ROIs were then used to quantify mean intensity in selected channels with the “quantify” function in 3D Manager.

For quantification in Figures 4B and 4C, H–I, 7A, and S4D, the image z stack was first max-intensity or sum-intensity projected. Then the nuclear ROIs were generated from the Hoechst channel by Gaussian blur, thresholding with the Otsu method, hole filling by the “Fill holes” function, and size selected with the “Analyze particle” function to only include particles larger than 50 μm^2^. Then the ROIs were used to quantify mean intensity and standard deviation in selected channels with the “Measure” function. For nuclear speckle analysis, a binary mask for the nuclei was first generated, then the channels of interest were multiplied to the mask to isolate nuclei. A duplicated SC35 channel was then used to generate nuclear speckle ROIs by Gaussian blur, local thresholding with the Bernsen method, size selected with the “Analyze particle” function to only include particles larger than 0.4 μm^2^. Then the ROIs were used to quantify mean intensity, area, and circularity in original SC35 channel with the “Measure” function.

### 4sU-spike-in RNA production using *in vitro* transcription

4sU-labeled or unlabeled spike-in RNA was produced according to a protocol described previously with modifications.^69^ Briefly, ERCC RNA Spike-In Mix (Thermo Fisher, 4456740) was reverse transcribed using AMV-RT (Promega, cat #M5108) and random 9-mer primer (IDT). Amplicons containing the T7 promoter were amplified using primers in Table S1 and Q5 2X master mix. *In vitro* transcription was performed using T7 RNA polymerase (NEB, M0251L) and 5 mM each of ATP, GTP, and CTP. For labeled spike-in, 4.5 mM UTP and 0.5 mM 4-thio-UTP (Jena Bioscience, NU-1156S) were used; for unlabeled spike-in, 5 mM UTP was used. Template DNA was then digested with TURBO DNase. RNA was then purified by phenol:chloroform extraction and cleaned up with Zymo RNA clean and concentrator −5 to remove unincorporated 4-thio-UTP. An equal mass of 3 labeled spike-in RNAs and 3 unlabeled spike-in RNAs was pooled and used for 4sU-seq.

### 4sU-seq

Six million A549 WT or RLKO cells were seeded in 15 cm tissue culture dishes and cultured overnight before transfection. Then, cells were then transfected with poly(I:C) for 4 h, followed by pulse labeling with 500 μM 4sU dissolved in DMEM media supplemented with 10% FBS for 10 min. The plates were washed once with PBS, then lysed with 2 mL TRIzol. RNA was extracted from the TRIzol lysate, followed by isopropanol precipitation. 100 μg or 300 μg (WT with poly(I:C)) total RNA was combined with an equal amount of pooled spike-in RNA and was biotinylated with HPDP-biotin (Thermo Scientific cat # PI21341, 50 μg per 25 μg of RNA) for 2 h, followed by two chloroform extractions and isopropanol precipitation. The RNA samples were denatured at 65°C for 5 min, then incubated with Dynabeads MyOne Streptavidin C1 magnetic beads (Thermo Fisher, cat # 65002) that had been previously blocked with glycogen for 1 h. The beads were washed twice with wash buffer (100 mM Tris-HCl, 10 mM EDTA, 1 M sodium chloride, and 0.1% Tween 20) at 65°C then twice at room temperature. To increase the stringency of the washes, the beads were further washed with TE buffer (10 mM Tris-HCl pH 7.4, 1 mM EDTA) twice at 55°C.^70^ 4sU-RNA was then eluted from the beads twice with 100 μL of 100 mM DTT, and the combined eluent was precipitated with isopropanol.

The sequencing library was constructed using KAPA Stranded RNA-Seq Kit with RiboErase (HMR) (cat # KK8484, 07962304001) and KAPA Single-Indexed Adapter Kit (Roche, cat # KK8700, 08005699001) following manufacturer’s instructions, with the following modifications: Briefly, 60 ng–100 ng RNA was used as input without rRNA depletion and fragmented at 85°C for 6 min; the PCR cycle numbers were determined empirically by qPCR. The library was submitted to QB3-Berkeley Genomics Sequencing Core for QC and sequencing on a NovaSeq 6000 S1 chip.

### Analysis of published RNA-seq datasets

Published total RNA-seq datasets for mock- or poly(I:C)-transfected A549 cells (GEO: GSE124144)^2^ were used to as a proxy to assign the dominant transcript for each gene. The raw fastq files were first pre-processed using HTStream (https://github.com/s4hts/HTStream) then pseudo-aligned and quantified using Kallisto.^71^ For each gene, the highest expressed transcript (measured by transcript-per-million, TPM, first criteria) with the longest transcript length (second criteria) was selected as the dominant transcript. Only genes on the main nuclear chromosomes (i.e., chr1–22, chrX and chrY) and the corresponding dominant transcript with average TPM > 0 are included.

### 4sU-seq data processing

The raw reads were pre-processed using HTStream v1.3.2 (https://github.com/s4hts/HTStream), which included the separation of rRNA and non-rRNA reads into separate files via HTStream SeqScreener. The rRNA reads were aligned to the rDNA repeat (GenBank Accession U13369.1), concatenated with ERCC sequences, and the non-rRNA reads were aligned to the human genome (hg38), concatenated with ERCC sequences, using STAR v2.6.1b.^56^ When aligning rRNA reads, options *–alignIntronMax 1* and *–alignSJD-BoverhangMin 999* were set to suppress gapped alignment. Aligned reads were counted using Rsubread (v2.16.1) FeatureCounts,^57^ and differential gene expression analysis was performed with limma-Voom v3.58.1, normalized to 4sU-labeled spike-in.^58^ A model of ~0+group+replicate is used to account for batch effects. Segments of pre-rRNA (i.e., 5′ ETS, 18S, ITS1, 5.8S, ITS2, 28S, 3′ ETS) were included in the differential expression analysis. Scaled, stranded BigWig files were generated with Deeptools bamCoverage function, using scaling factors generated in limma-Voom (proportional to the reciprocal of norm.factors × lib.sizes). BigWig files of biological replicates were averaged using Deeptools bigwigAverage function. For both operations, bin sizes were set to 20bp. The metagene coverage plots were generated using Deeptools^59^ using the coordinates of dominant transcripts defined in “Analysis of published RNA-seq datasets”. GO term analysis was performed with the topGO package (https://doi.org/10.18129/B9.bioc.topGO), using the Weight01 algorithm and Fisher’s exact test.

### CUT&RUN-sequencing

The CUT&RUN procedure was performed according to previous reports,^72,73^ with modifications. Briefly, 250,000 A549 WT or RNase L KO cells were seeded per well in a 12-well plate and cultured overnight before transfection. Then cells were then transfected with RNAiMAX alone or with 0.76 μg/mL poly(I:C) for 4 h The cells were permeabilize on plate with 500 μL Perm buffer (20 mM HEPES-KOH pH 7.5, 150 mM NaCl, 0.5 mM Spermidine (Sigma, S2626), 0.1% Triton X-100 (Sigma, X100), and proteinase inhibitor (Roche, 04693132001)) for 15 min at room temperature. The cells were washed again with Perm buffer then incubated with either anti-Rpb1 S5P CTD (4H8, Cell Signaling, cat #2629) at 1: 50 dilution, equal mass of mouse normal IgG (Santa Cruz, cat # sc-2025), or anti-Rpb1 S2P CTD (clone E1Z3G, Cell Signaling, cat # 13499) at 1: 50 dilution, in 200μL Perm buffer for 1 h at room temperature. The cells were then washed twice with Perm buffer and incubated with pAG-MNase (Cell Signaling, cat #40366) at a 1:33 dilution in 200 μL Perm buffer for 1 h at room temperature. The cells were then washed twice with Perm buffer and chilled on ice. Then, 225 μL ice-cold Perm buffer containing 5 mM CaCl_2_ was dispensed per well, and the plate was incubated in a refrigerator at 4°C for 30 min for targeted digestion. The reaction was halted by adding 75 μL 4× STOP solution (680 mM NaCl, 40 mM EDTA, 8 mM EGTA, 100 μg/mL RNase A (Invitrogen, cat # 12091021) and 0.1% Triton X-100, spiked with yeast genomic DNA (Cell Signaling, cat #40366) at 10 pg per sample for 4H8 and IgG, 30 pg per sample for E1Z3G). The plate was then incubated at 37°C for 30 min to facilitate fragment release. The supernatant was collected, and then 3 μL 20% SDS solution and 3.75 μL 20 mg/mL proteinase K solution were added. The mixture was then incubated at 50°C for 1 h. The DNA was extracted with phenol:chloroform.

The sequencing library was constructed with NEBNext Ultra II DNA Library Prep Kit (NEB, cat #E7645L) following a previous report^74^ with modifications. Briefly, equal volume of purified DNA fragments was used. End preparation was performed at 20°C for 30 min and then at 50°C for 60 min to prevent short fragment melting. Fragments were then ligated to adaptors from NEBNext Multiplex Oligos for Illumina (Unique Dual Index UMI Adaptors DNA Set 1, NEB, cat #E7395S) and cleaned up twice with 1.1X Ampure XP beads (Beckman Coutler, cat # A63880). The resulting ligated products were then PCR amplified for 10 cycles for 4H8 and E1Z3G, and 14 cycles for IgG samples, respectively. The cycle numbers were determined empirically by qPCR. The amplified libraries were double-sided size-selected with 0.6X and 1.1X Ampure XP beads and further cleaned up by 1 round with 1.1X beads for 4H8, E1Z3G, or 3 rounds for IgG samples, to remove adaptor dimers. The libraries were sequenced on AVITI platform (Element Biosciences) by UC Davis sequencing core.

### CUT&RUN data processing

Reads were trimmed with Trimmomatic v0.39^60^ and aligned to a concatenated human (hg38) and yeast (SacCer3) genome with bowtie 2 v2.5.2.^61^ The BAM files were then deduplicated with UMI_tools v1.1.5^63^ and optionally splitted into subnucleosomal (<120 bp) and nucleosomal (>120 bp) fractions using Deeptools (v3.5.5) alignmentSieve. The spike-in normalization factors were calculated by counting reads mapped to yeast genome using Samtools idxstats,^62^ followed by conversion to scaling factors that are proportional to the reciprocal to the spike-in read counts. BigWig files were generated with Deeptools bamCoverage function, using scaling factors generated above. BigWig files of biological replicates were averaged using Deeptools bigwigAverage function. For both operations, bin sizes were set to 20 bp. The metagene coverage plots were generated using Deeptools. Differential binding analysis was performed with DiffBind v3.12.0^64^ using the built-in spike-in normalization function in *dba.normalize*. A model of ~0+group+replicate is used to account for batch effects. For analysis in Figure 6, different windows were analyzed separately to avoid merging of regions by diffBind. For elongation index calculation, FeatureCounts was used to count reads mapped to either TSS (−500 bp to +250 bp) or gene body (+250 bp to TES). One pseudocount was then added to all regions. Then all samples were normalized using the spike-in scaling factors generated above. Only genes with >16 counts/kb at both the TSS and the gene body were used in subsequent calculations. Elongation index was averaged by taking the geometric mean of 3 replicates, followed by logarithmic conversion.

### Quantitative reverse transcription PCR (RT-qPCR)

500,000 A549 WT or RLKO cells per well were seeded in 6-well plates the day before either mock-transfecting or transfecting with 0.76 μg/ml poly(I:C), in the absence or in the presence of 1X brefeldin A (eBiosciences, cat # 00–4506-51) when indicated, for 3 h. RNA samples were prepared by TRIzol extraction. They were then treated with Turbo DNase (Thermo Fisher, cat # AM2238) for 30 min at 37°C and re-purified by phenol:chloroform extraction. 500 ng to 1 μg RNA was used for reverse transcription using AMV-RT (Promega, cat #M5108) and random 9-mer primer (IDT). cDNA was then quantified by qPCR using iTaq Universal SYBR Mastermix (Bio-Rad, cat # 1725125) and the indicated primer pairs. Transcript levels were normalized to 18S.

For Figure S2B, an equal volume of 4sU RNA eluent was directly reverse-transcribed using AMV-RT (Promega) and a random 9-mer primer (IDT), and quantified as above with the indicated primer pairs. Transcript levels were normalized to spike-in.

For Figure 7B, 1μg RNA was treated with Turbo DNase for 15 min at 37°C and inactivated at 70°C in the presence of 15 mM EDTA. The treated RNA was diluted with DEPC water to 50 μL and 4 μL was used for reverse transcription using AMV-RT and random 9-mer primer. cDNA was then quantified by qPCR with iTaq Universal SYBR Mastermix.

### Western blotting

To prepare whole-cell lysates for evaluating RNase L knockdown, murine iBMDMs transfected with RNase L targeting siRNAs (Horizon, cat # L-043480–00-0010) were washed with cold DPBS (Gibco) followed by lysis with radioimmunoprecipitation assay (RIPA) lysis buffer (50 mM Tris HCl, 150 mM NaCl, 1.0% [vol/vol] NP-40, 0.5% [wt/vol] sodium deoxycholate, 1.0 mM EDTA, and 1% [wt/vol] SDS, Halt Protease and Phosphatase Inhibitor Cocktail (Thermo Fisher, cat # PI78440). Cell lysates were vortexed briefly, rotated at 4°C for 15 min, and then clarified by centrifugation at 21,000 × *g* in a tabletop centrifuge at 4°C for 10 min to remove debris. 30 μg of whole-cell lysate were resolved on 4%–15% mini-PROTEAN TGX gels (Bio-Rad). Transfers to polyvinylidene difluoride (PVDF) membranes (Bio-Rad) were done with the Trans-Blot Turbo transfer system (Bio-Rad). Blots were incubated in 5% milk in TBS with 0.1% Tween 20 (TBS-T) to block, followed by incubation with primary antibodies against anti-RNase L (Abcam, ab191392, 1:10,000) and anti-GAPDH antibody (abcam ab8245, 1;5,000). Washes were carried out with TBS-T. Blots were then incubated with HRP-conjugated secondary antibodies (Southern Biotechnology, 1:5,000). Washed blots were incubated with Clarity Western ECL substrate (Bio-Rad) for 5 min and visualized with a ChemiDoc imager (Bio-Rad). Band intensity was analyzed with ImageJ.^66^

## QUANTIFICATION AND STATISTICAL ANALYSIS

All statistical analyses were performed with GraphPad Prism 10 and RStudio. For limma-Voom analysis for 4sU-seq, and DiffBind analysis for CUT&RUN-seq, adjusted *p* value or False discovery rate (FDR) is corrected with the Benjamini-Hochberg (BH) method. All statistical method details and the *p* value notation are included in figure legends.

## Supplementary Material

1

2

3

4

5

6

Supplemental information can be found online at https://doi.org/10.1016/j.celrep.2026.117028.

## Figures and Tables

**KEY RESOURCES TABLE:** 

REAGENT or RESOURCE	SOURCE	IDENTIFIER

Antibodies		

mouse anti-NS3 antibody (Clone E1D8)	Balsitis et al.^53^	N/A
POLR1A (D6S6S) Rabbit mAb	Cell Signaling Technology	Cat# 24799; RRID:AB_2798884
Nucleophosmin antibody [FC82291]	Abcam	Cat# ab10530; RRID:AB_297271
Rpb1 NTD (D8L4Y)	Cell Signaling Technology	Cat# 14958; RRID:AB_2687876
Rpb1 S5P CTD (ab5131) (for immunofluorescence)	Abcam	Cat# ab5131; RRID:AB_449369
Rpb1 S5P CTD (3E8) (for immunofluorescence)	Active Motif	Cat# 61085; RRID:AB_2687451
Rpb1 S5P CTD (4H8) (for CUT&RUN)	Cell Signaling Technology	Cat# 2629; RRID:AB_2167468
Rpb1 S2P CTD (E1Z3G) (for CUT&RUN)	Cell Signaling Technology	Cat# 13499; RRID:AB_2798238
Rpb1 S2P CTD (ab5095) (for immunofluorescence)	Abcam	Cat# ab5095; RRID:AB_304749
Mouse Anti-SC35 Monoclonal Antibody	Abcam	Cat# ab11826; RRID:AB_298608
Recombinant Anti-RNase L antibody [EPR15894]	Abcam	Cat# ab191392; RRID:AB_2916269
GAPDH antibody [6C5]	Abcam	Cat# ab8245; RRID:AB_2107448
goat anti-rabbit IgG Alexa Fluor Plus 488	Thermo Fisher Scientific	Cat# A32723; RRID:AB_2633275
goat anti-mouse IgG Alexa Fluor 546	Thermo Fisher Scientific	Cat# A-11035; RRID:AB_2534093
donkey anti-rat IgG Alexa Fluor Plus 488	Thermo Fisher Scientific	Cat# A48269; RRID:AB_2893137
goat anti-rabbit IgG Alexa Fluor 647	Thermo Fisher Scientific	Cat# A27040; RRID:AB_2536101
Goat Anti-Rabbit IgG-HRP	SouthernBiotech	Cat# 4030-05; RRID:AB_2687483
Mouse normal IgG	Santa Cruz Biotechnology	Cat# sc-2025; RRID:AB_737182

Bacterial and virus strains		

Zika virus (Nica 2-16)	Tabata et al^54^	GenBank accession KX421194.1

Chemicals, peptides, and recombinant proteins		

4-thiouridine (4SU)	Sigma Aldrich	T4509-100MG
HPDP-Biotin	Thermo Fisher Scientific	PI21341
pAG-MNase	Cell Signaling	40366S
proteinase K	Cell Signaling	10012S
RNase A	Thermo Fisher Scientific	EN0531
4-Thio-UTP	Jena Bioscience	NU-1156S
T7 RNA polymerase	NEB	M0251L (25,000U)
CFDA-SE	Cayman Chemical	14456
5-Ethynyl-uridine	Lumiprobe	2439-500mg
5-Ethynyl-uridine	Click Chemistry Tools	cat # CCT-1261-500
Sulfo-Cyanine5-Azide	Lumiprobe	A3330
Alexa Fluor 488 Azide	Thermo Fisher Scientific	A10266
Ibetazol	Axon MedChem	Axon 4300
In-Fusion^®^ snap assembly (master mix)	Clontech (TaKaRa)	638943
TURBO DNase	Thermo Fisher Scientific	AM2239 (5,000U)
AMV Reverse Transcriptase	Promega	M5101 (300U)
cOmplete EDTA-free protease inhibitor cocktail	Roche	5056489001
Halt Protease and Phosphatase Inhibitor Cocktail (100X)	Thermo Fisher Scientific	78440
Poly(I:C) (HMW)	InVivoGen	tlrl-pic, tlrl-picf
Lipofectamine RNAiMAX	Thermo Fisher Scientific	13778150
Brefeldin A Solution (1000X)	Thermo Fisher Scientific	00-4506-51
Janelia Fluor 646 HaloTag Ligand	Promega	GA1121

Critical commercial assays		

KAPA Stranded RNA-Seq Kit with RiboErase (HMR)	Roche	KK8484 07962304001
KAPA Single-Indexed Adapter Kit	Roche	KK8700 08005699001
ERCC RNA Spike-In Mix	Thermo Fisher Scientific	4456740
NEBNext Ultra II DNA Library Prep Kit	NEB	cat #E7645L
NEBNext Multiplex Oligos for Illumina (Unique Dual Index UMI Adaptors DNA Set 1)	NEB	cat #E7395S
Universal Mycoplasma Detection Kit	ATCC	50189644FP
iTaq universal SYBR green supermix	Bio-Rad	1725121
Q5 Hot Start High-Fidelity 2X Master Mix	NEB	M0494S

Deposited data		

Raw and processed 4sU-seq data	This study	GEO: GSE298174
Raw and processed S5P Pol II CUT&RUN-seq data	This study	GEO: GSE298175
Raw and processed S5P Pol II CUT&RUN-seq data	This study	GEO: GSE313712
Total RNA-seq for mock- or poly(I:C)-transfected A549 cells	Burke et al^2^	GEO: GSE124144

Experimental models: Cell lines		

A549 WT cells	Burke et al.^2^	N/A
A549 RLKO cells	Burke et al.^2^	N/A
A549 RLKO cells reconstituted with 3xflag-RNase L (A549 RLKO-RL)	Burke et al.^2^	N/A
A549 RLKO cells reconstituted with catalytic dead 3xflag-RNase L (R667A) (A549 RLKO-CM)	Burke et al.^2^	N/A
Murine immortal bone-marrow-derived macrophages (iBMDM)	Robinson et al.^55^	N/A

Oligonucleotides		

2-5A (pA(2-5A)4)	IDT	N/A
ON-TARGETplus Non-targeting Control Pool	Dharmacon	D-001810-10-05
SMARTPool: ON-TARGETplus Mouse RNASEL siRNA,10nM	Dharmacon	L-043480-00-0010
Table S1	This paper	N/A

Recombinant DNA		

pLJM1-Halo-RNaseL	This study	Addgene: 250856
pLJM1-CRS-Halo-RNaseL	This study	Addgene: 250857
pLJM1-CRS-Halo-NLS	This study	Addgene: 250859
pLJM1-Halo-NLS	This study	Addgene: 250858

Software and algorithms		

HTStream		RRID:SCR_018354
STAR	Dobin et al.^56^	RRID:SCR_004463
FeatureCounts	Liao et al.^57^	RRID:SCR_012919
Limma-voom	Ritchie et al.^58^	RRID:SCR_010943
topGO	https://doi.org/10.18129/B9.bioc.topGO	RRID:SCR_014798
Deeptools	Ramirez et al.^59^	RRID:SCR_016366
Trimmomatic	Bolger et al.^60^	RRID:SCR_011848
Bowtie 2	Langmead and Salzberg.^61^	RRID:SCR_016368
SAMTOOLS	Li et al.^62^	RRID:SCR_002105
UMI-tools	Smith et al.^63^	RRID:SCR_017048
DiffBind	Ross-Innes et al.^64^	RRID:SCR_012918
FIJI	Schindelin et al.^65^	RRID:SCR_002285
ImageJ	Schneider et al.^66^	RRID:SCR_003070
Prism 10 v10.3.0	GraphPad	N/A
R	www.r-project.org	N/A
RStudio	www.rstudio.com	N/A

Other		

Dynabeads MyOne Streptavidin C1	Thermo Fisher Scientific	65002

## Highlights

- Cells with activated RNase L have broadly reduced levels of nascent RNA in the nucleus
- Reduced nascent RNA is due to repression of transcription by RNA polymerases I, II, and III
- Transcriptional repression occurs primarily at the stage of elongation
- RNA decay-to-transcription feedback involves protein transport by importin β

## References

[1] Karasik A, and Guydosh NR (2024). The Unusual Role of Ribonuclease L in Innate Immunity. Wiley Interdiscip. Rev. RNA 15, e1878. 10.1002/wrna.1878. 39727035 PMC11672174

[2] Burke JM, Moon SL, Matheny T, and Parker R (2019). RNase L Reprograms Translation by Widespread mRNA Turnover Escaped by Antiviral mRNAs. Mol. Cell 75, 1203–1217.e5. 10.1016/j.molcel.2019.07.029. 31494035 PMC6754297

[3] Donovan J, Rath S, Kolet-Mandrikov D, and Korennykh A (2017). Rapid RNase L-driven arrest of protein synthesis in the dsRNA response without degradation of translation machinery. RNA 23, 1660–1671. 10.1261/rna.062000.117. 28808124 PMC5648034

[4] Rath S, Prangley E, Donovan J, Demarest K, Wingreen NS, Meir Y, and Korennykh A (2019). Concerted 2–5A-Mediated mRNA Decay and Transcription Reprogram Protein Synthesis in the dsRNA Response. Mol. Cell 75, 1218–1228.e6. 10.1016/j.molcel.2019.07.027. 31494033 PMC6754276

[5] Chitrakar A, Rath S, Donovan J, Demarest K, Li Y, Sridhar RR, Weiss SR, Kotenko SV, Wingreen NS, and Korennykh A (2019). Real-time 2–5A kinetics suggest that interferons beta and lambda evade global arrest of translation by RNase L. Proc. Natl. Acad. Sci. USA 116, 2103–2111. 10.1073/pnas.1818363116. PMC6369740 30655338

[6] Burke JM, Gilchrist AR, Sawyer SL, and Parker R (2021). RNase L limits host and viral protein synthesis via inhibition of mRNA export. Sci. Adv. 7, eabh2479. 10.1126/sciadv.abh2479. 34088676 PMC8177694

[7] Burke JM, Ripin N, Ferretti MB, St Clair LA, Worden-Sapper ER, Salgado F, Sawyer SL, Perera R, Lynch KW, and Parker R (2022). RNase L activation in the cytoplasm induces aberrant processing of mRNAs in the nucleus. PLoS Pathog. 18, e1010930. 10.1371/journal.ppat.1010930. 36318584 PMC9651596

[8] Karasik A, Lorenzi HA, DePass AV, and Guydosh NR (2024). Endonucleolytic RNA cleavage drives changes in gene expression during the innate immune response. Cell Rep. 43, 114287. 10.1016/j.celrep.2024.114287. 38823018 PMC11251458

[9] Lee D, Le Pen J, Yatim A, Dong B, Aquino Y, Ogishi M, Pescarmona R, Talouarn E, Rinchai D, Zhang P, (2023). Inborn errors of OAS-RNase L in SARS-CoV-2-related multisystem inflammatory syndrome in children. Science 379, eabo3627. 10.1126/science.abo3627. 36538032 PMC10451000

[10] Malathi K, Dong B, Gale M Jr., and Silverman RH (2007). Small self-RNA generated by RNase L amplifies antiviral innate immunity. Nature 448, 816–819. 10.1038/nature06042. 17653195 PMC3638316

[11] Xi J, Snieckute G, Martínez JF, Arendrup FSW, Asthana A, Gaughan C, Lund AH, Bekker-Jensen S, and Silverman RH (2024). Initiation of a ZAKα-dependent ribotoxic stress response by the innate immunity endoribonuclease RNase L. Cell Rep. 43, 113998. 10.1016/j.celrep.2024.113998. 38551960 PMC11090160

[12] Yang K, Dong B, Asthana A, Silverman RH, and Yan N (2024). RNA helicase SKIV2L limits antiviral defense and autoinflammation elicited by the OAS-RNase L pathway. EMBO J. 43, 3876–3894. 10.1038/s44318-024-00187-1. 39112803 PMC11405415

[13] Banerjee S, Chakrabarti A, Jha BK, Weiss SR, and Silverman RH (2014). Cell-type-specific effects of RNase L on viral induction of beta interferon. mBio 5, e00856–14. 10.1128/mBio.00856-14. 24570368 PMC3940032

[14] Iordanov MS, Paranjape JM, Zhou A, Wong J, Williams BR, Meurs EF, Silverman RH, and Magun BE (2000). Activation of p38 mitogen-activated protein kinase and c-Jun NH(2)-terminal kinase by double-stranded RNA and encephalomyocarditis virus: involvement of RNase L, protein kinase R, and alternative pathways. Mol. Cell Biol. 20, 617–627. 10.1128/MCB.20.2.617-627.2000. 10611240 PMC85147

[15] Lee YJ, and Glaunsinger BA (2009). Aberrant herpesvirus-induced polyadenylation correlates with cellular messenger RNA destruction. PLoS Biol. 7, e1000107. 10.1371/journal.pbio.1000107. 19468299 PMC2680333

[16] Kumar GR, and Glaunsinger BA (2010). Nuclear import of cytoplasmic poly(A) binding protein restricts gene expression via hyperadenylation and nuclear retention of mRNA. Mol. Cell Biol. 30, 4996–5008. 10.1128/MCB.00600-10. 20823266 PMC2953054

[17] Kumar GR, Shum L, and Glaunsinger BA (2011). Importin alpha-mediated nuclear import of cytoplasmic poly(A) binding protein occurs as a direct consequence of cytoplasmic mRNA depletion. Mol. Cell Biol. 31, 3113–3125. 10.1128/MCB.05402-11. 21646427 PMC3147611

[18] Gaglia MM, Covarrubias S, Wong W, and Glaunsinger BA (2012). A common strategy for host RNA degradation by divergent viruses. J. Virol. 86, 9527–9530. 10.1128/JVI.01230-12. 22740404 PMC3416159

[19] Abernathy E, Gilbertson S, Alla R, and Glaunsinger B (2015). Viral Nucleases Induce an mRNA Degradation-Transcription Feedback Loop in Mammalian Cells. Cell Host Microbe 18, 243–253. 10.1016/j.chom.2015.06.019. 26211836 PMC4538998

[20] Gilbertson S, Federspiel JD, Hartenian E, Cristea IM, and Glaunsinger B (2018). Changes in mRNA abundance drive shuttling of RNA binding proteins, linking cytoplasmic RNA degradation to transcription. eLife 7, e37663. 10.7554/eLife.37663. 30281021 PMC6203436

[21] Hartenian E, Gilbertson S, Federspiel JD, Cristea IM, and Glaunsinger BA (2020). RNA decay during gammaherpesvirus infection reduces RNA polymerase II occupancy of host promoters but spares viral promoters. PLoS Pathog. 16, e1008269. 10.1371/journal.ppat.1008269. 32032393 PMC7032723

[22] Thomas MP, Liu X, Whangbo J, McCrossan G, Sanborn KB, Basar E, Walch M, and Lieberman J (2015). Apoptosis Triggers Specific, Rapid, and Global mRNA Decay with 3’ Uridylated Intermediates Degraded by DIS3L2. Cell Rep. 11, 1079–1089. 10.1016/j.celrep.2015.04.026. 25959823 PMC4862650

[23] Liu X, Fu R, Pan Y, Meza-Sosa KF, Zhang Z, and Lieberman J (2018). PNPT1 Release from Mitochondria during Apoptosis Triggers Decay of Poly(A) RNAs. Cell 174, 187–201.e12. 10.1016/j.cell.2018.04.017. 29779946

[24] Duncan-Lewis C, Hartenian E, King V, and Glaunsinger BA (2021). Cytoplasmic mRNA decay represses RNA polymerase II transcription during early apoptosis. eLife 10, e58342. 10.7554/eLife.58342. 34085923 PMC8192121

[25] Siddiqui MA, and Malathi K (2012). RNase L induces autophagy via c-Jun N-terminal kinase and double-stranded RNA-dependent protein kinase signaling pathways. J. Biol. Chem. 287, 43651–43664. 10.1074/jbc.M112.399964. 23109342 PMC3527951

[26] Watkins JM, and Burke JM (2024). RNase L-induced bodies sequester subgenomic flavivirus RNAs to promote viral RNA decay. Cell Rep. 43, 114694. 10.1016/j.celrep.2024.114694. 39196777 PMC11957735

[27] Whelan JN, Li Y, Silverman RH, and Weiss SR (2019). Zika Virus Production Is Resistant to RNase L Antiviral Activity. J. Virol. 93, e00313–19. 10.1128/JVI.00313-19. 31142667 PMC6675901

[28] Whelan JN, Parenti NA, Hatterschide J, Renner DM, Li Y, Reyes HM, Dong B, Perez ER, Silverman RH, and Weiss SR (2021). Zika virus employs the host antiviral RNase L protein to support replication factory assembly. Proc. Natl. Acad. Sci. USA 118, e2101713118. 10.1073/pnas.2101713118. 34031250 PMC8179202

[29] Slomnicki LP, Chung DH, Parker A, Hermann T, Boyd NL, and Hetman M (2017). Ribosomal stress and Tp53-mediated neuronal apoptosis in response to capsid protein of the Zika virus. Sci. Rep. 7, 16652. 10.1038/s41598-017-16952-8. 29192272 PMC5709411

[30] Berry S, Müller M, Rai A, and Pelkmans L (2022). Feedback from nuclear RNA on transcription promotes robust RNA concentration homeostasis in human cells. Cell Syst. 13, 454–470.e15. 10.1016/j.cels.2022.04.005. 35613616

[31] Rädle B, Rutkowski AJ, Ruzsics Z, Friedel CC, Koszinowski UH, and Dölken L (2013). Metabolic labeling of newly transcribed RNA for high resolution gene expression profiling of RNA synthesis, processing and decay in cell culture. J. Vis. Exp 50195. 10.3791/50195. 23963265 PMC3854562

[32] Andersen JB, Mazan-Mamczarz K, Zhan M, Gorospe M, and Hassel BA (2009). Ribosomal protein mRNAs are primary targets of regulation in RNase-L-induced senescence. RNA Biol. 6, 305–315. 10.4161/rna.6.3.8526. 19411840 PMC2752476

[33] Shore D, and Albert B (2022). Ribosome biogenesis and the cellular energy economy. Curr. Biol. 32, R611–R617. 10.1016/j.cub.2022.04.083. 35728540

[34] Niu TK, Pfeifer AC, Lippincott-Schwartz J, and Jackson CL (2005). Dynamics of GBF1, a Brefeldin A-sensitive Arf1 exchange factor at the Golgi. Mol. Biol. Cell 16, 1213–1222. 10.1091/mbc.e04-07-0599. 15616190 PMC551486

[35] Cusic R, and Burke JM (2024). Condensation of RNase L promotes its rapid activation in response to viral infection in mammalian cells. Sci. Signal. 17, eadi9844. 10.1126/scisignal.adi9844. 38771918 PMC11391522

[36] Zhu Z, Liu J, Feng H, Zhang Y, Huang R, Pan Q, Nan J, Miao R, and Cheng B (2022). Overcoming the cytoplasmic retention of GDOWN1 modulates global transcription and facilitates stress adaptation. eLife 11, e79116. 10.7554/eLife.79116. 36476745 PMC9728996

[37] Hoboth P, Sztacho M, and Hozák P (2024). Nuclear patterns of phosphatidylinositol 4,5- and 3,4-bisphosphate revealed by super-resolution microscopy differ between the consecutive stages of RNA polymerase II transcription. FEBS J. 291, 4240–4264. 10.1111/febs.17136. 38734927

[38] Fan Z, Devlin JR, Hogg SJ, Doyle MA, Harrison PF, Todorovski I, Cluse LA, Knight DA, Sandow JJ, Gregory G, (2020). CDK13 cooperates with CDK12 to control global RNA polymerase II processivity. Sci. Adv. 6, eaaz5041. 10.1126/sciadv.aaz5041. 32917631 PMC7190357

[39] Gu B, Eick D, and Bensaude O (2013). CTD serine-2 plays a critical role in splicing and termination factor recruitment to RNA polymerase II in vivo. Nucleic Acids Res. 41, 1591–1603. 10.1093/nar/gks1327. 23275552 PMC3561981

[40] Harlen KM, and Churchman LS (2017). The code and beyond: transcription regulation by the RNA polymerase II carboxy-terminal domain. Nat. Rev. Mol. Cell Biol. 18, 263–273. 10.1038/nrm.2017.10. 28248323

[41] Vercruysse T, Vanstreels E, Jacquemyn M, Boland S, Kilonda A, Allasia S, Vandecaetsbeek I, Klaassen H, Versele M, Chaltin P, (2024). Ibetazol, a novel inhibitor of importin β1-mediated nuclear import. Commun. Biol. 7, 1560. 10.1038/s42003-024-07237-8. 39580542 PMC11585640

[42] Aoi Y, Iravani L, Mroczek IC, Gold S, Howard BC, and Shilatifard A (2025). SPT5 regulates RNA polymerase II stability via Cullin 3-ARMC5 recognition. Sci. Adv. 11, eadt5885. 10.1126/sciadv.adt5885. 39854452 PMC11758996

[43] Blears D, Lou J, Fong N, Mitter R, Sheridan RM, He D, Dirac-Svejstrup AB, Bentley D, and Svejstrup JQ (2024). Redundant pathways for removal of defective RNA polymerase II complexes at a promoter-proximal pause checkpoint. Mol. Cell 84, 4790–4807.e11. 10.1016/j.molcel.2024.10.012. 39504960 PMC11663130

[44] Cacioppo R, Gillis A, Shlamovitz I, Zeller A, Castiblanco D, Crisp A, Haworth B, Arabiotorre A, Abyaneh P, Bao Y, (2024). CRL3^ARMC5^ ubiquitin ligase and Integrator phosphatase form parallel mechanisms to control early stages of RNA Pol II transcription. Mol. Cell 84, 4808–4823.e13. 10.1016/j.molcel.2024.11.024. 39667934 PMC7617427

[45] Potapova TA, Unruh JR, Conkright-Fincham J, Banks CAS, Florens L, Schneider DA, and Gerton JL (2023). Distinct states of nucleolar stress induced by anticancer drugs. eLife 12. 10.7554/eLife.88799. PMC10723795 38099650

[46] Williams TD, Michalak EM, Carey KT, Lam EYN, Anderson A, Griesbach E, Chan YC, Papasaikas P, Tan VWT, Ngo L, (2025). mRNA export factors store nascent transcripts within nuclear speckles as an adaptive response to transient global inhibition of transcription. Mol. Cell 85, 117–131.e7. 10.1016/j.molcel.2024.12.008. 39753105

[47] Kim J, Han KY, Khanna N, Ha T, and Belmont AS (2019). Nuclear speckle fusion via long-range directional motion regulates speckle morphology after transcriptional inhibition. J. Cell Sci. 132, jcs226563. 10.1242/jcs.226563. 30858197 PMC6503955

[48] Decker CJ, Burke JM, Mulvaney PK, and Parker R (2022). RNA is required for the integrity of multiple nuclear and cytoplasmic membrane-less RNP granules. EMBO J. 41, e110137. 10.15252/embj.2021110137. 35355287 PMC9058542

[49] Aprile-Garcia F, Tomar P, Hummel B, Khavaran A, and Sawarkar R (2019). Nascent-protein ubiquitination is required for heat shock-induced gene downregulation in human cells. Nat. Struct. Mol. Biol. 26, 137–146. 10.1038/s41594-018-0182-x. 30723328

[50] Bulut-Karslioglu A, Macrae TA, Oses-Prieto JA, Covarrubias S, Percharde M, Ku G, Diaz A, McManus MT, Burlingame AL, and Ramalho-Santos M (2018). The Transcriptionally Permissive Chromatin State of Embryonic Stem Cells Is Acutely Tuned to Translational Output. Cell Stem Cell 22, 369–383.e8. 10.1016/j.stem.2018.02.004. 29499153 PMC5836508

[51] Takenaka Y, Yamada A, Tomioka Y, Akiyama Y, and Ivanov P (2025). RNase L Produces tRNA-derived RNAs that Contribute to Translation Inhibition. RNA 31, 961–972. 10.1261/rna.080419.125. 40233983 PMC12170186

[52] Huai W, Yang K, Xing C, Song K, Lyu H, Williams NS, Wu J, and Yan N (2025). OAS cross-activates RNase L intercellularly through cell-to-cell transfer of 2–5A to spread innate immunity. Immunity 58, 797–810.e6. 10.1016/j.immuni.2025.01.016. 40010341 PMC11981853

[53] Balsitis SJ, Coloma J, Castro G, Alava A, Flores D, McKerrow JH, Beatty PR, and Harris E (2009). Tropism of dengue virus in mice and humans defined by viral nonstructural protein 3-specific immunostaining. Am. J. Trop. Med. Hyg. 80, 416–424. 19270292

[54] Tabata T, Petitt M, Puerta-Guardo H, Michlmayr D, Wang C, Fang-Hoover J, Harris E, and Pereira L (2016). Zika Virus Targets Different Primary Human Placental Cells, Suggesting Two Routes for Vertical Transmission. Cell Host Microbe 20, 155–166. 10.1016/j.chom.2016.07.002. 27443522 PMC5257282

[55] Robinson EK, Jagannatha P, Covarrubias S, Cattle M, Smaliy V, Safavi R, Shapleigh B, Abu-Shumays R, Jain M, Cloonan SM, (2021). Inflammation drives alternative first exon usage to regulate immune genes including a novel iron-regulated isoform of *Aim2*. eLife 10, e69431. 10.7554/eLife.69431. 34047695 PMC8260223

[56] Dobin A, Davis CA, Schlesinger F, Drenkow J, Zaleski C, Jha S, Batut P, Chaisson M, and Gingeras TR (2013). STAR: ultrafast universal RNA-seq aligner. Bioinformatics 29, 15–21. 10.1093/bioinformatics/bts635. 23104886 PMC3530905

[57] Liao Y, Smyth GK, and Shi W (2014). featureCounts: an efficient general purpose program for assigning sequence reads to genomic features. Bioinformatics 30, 923–930. 10.1093/bioinformatics/btt656. 24227677

[58] Ritchie ME, Phipson B, Wu D, Hu Y, Law CW, Shi W, and Smyth GK (2015). limma powers differential expression analyses for RNA-sequencing and microarray studies. Nucleic Acids Res. 43, e47. 10.1093/nar/gkv007. 25605792 PMC4402510

[59] Ramírez F, Ryan DP, Grüning B, Bhardwaj V, Kilpert F, Richter AS, Heyne S, Dündar F, and Manke T (2016). deepTools2: a next generation web server for deep-sequencing data analysis. Nucleic Acids Res. 44, W160–W165. 10.1093/nar/gkw257. 27079975 PMC4987876

[60] Bolger AM, Lohse M, and Usadel B (2014). Trimmomatic: a flexible trimmer for Illumina sequence data. Bioinformatics 30, 2114–2120. 10.1093/bioinformatics/btu170. 24695404 PMC4103590

[61] Langmead B, and Salzberg SL (2012). Fast gapped-read alignment with Bowtie 2. Nat. Methods 9, 357–359. 10.1038/nmeth.1923. 22388286 PMC3322381

[62] Li H, Handsaker B, Wysoker A, Fennell T, Ruan J, Homer N, Marth G, Abecasis G, and Durbin R; 1000 Genome Project Data Processing Subgroup (2009). The Sequence Alignment/Map format and SAMtools. Bioinformatics 25, 2078–2079. 10.1093/bioinformatics/btp352. 19505943 PMC2723002

[63] Smith T, Heger A, and Sudbery I (2017). UMI-tools: modeling sequencing errors in Unique Molecular Identifiers to improve quantification accuracy. Genome Res. 27, 491–499. 10.1101/gr.209601.116. 28100584 PMC5340976

[64] Ross-Innes CS, Stark R, Teschendorff AE, Holmes KA, Ali HR, Dunning MJ, Brown GD, Gojis O, Ellis IO, Green AR, (2012). Differential oestrogen receptor binding is associated with clinical outcome in breast cancer. Nature 481, 389–393. 10.1038/nature10730. 22217937 PMC3272464

[65] Schindelin J, Arganda-Carreras I, Frise E, Kaynig V, Longair M, Pietzsch T, Preibisch S, Rueden C, Saalfeld S, Schmid B, (2012). Fiji: an open-source platform for biological-image analysis. Nat. Methods 9, 676–682. 10.1038/nmeth.2019. 22743772 PMC3855844

[66] Schneider CA, Rasband WS, and Eliceiri KW (2012). NIH Image to ImageJ: 25 years of image analysis. Nat. Methods 9, 671–675. 10.1038/nmeth.2089. 22930834 PMC5554542

[67] Sancak Y, Peterson TR, Shaul YD, Lindquist RA, Thoreen CC, Bar-Peled L, and Sabatini DM (2008). The Rag GTPases bind raptor and mediate amino acid signaling to mTORC1. Science 320, 1496–1501. 10.1126/science.1157535. 18497260 PMC2475333

[68] Chiu J, March PE, Lee R, and Tillett D (2004). Site-directed, Ligase-Independent Mutagenesis (SLIM): a single-tube methodology approaching 100% efficiency in 4 h. Nucleic Acids Res. 32, e174. 10.1093/nar/gnh172. 15585660 PMC535700

[69] Schwalb B, Michel M, Zacher B, Frühauf K, Demel C, Tresch A, Gagneur J, and Cramer P (2016). TT-seq maps the human transient transcriptome. Science 352, 1225–1228. 10.1126/science.aad9841. 27257258

[70] Li HB, Tong J, Zhu S, Batista PJ, Duffy EE, Zhao J, Bailis W, Cao G, Kroehling L, Chen Y, (2017). m^6^A mRNA methylation controls T cell homeostasis by targeting the IL-7/STAT5/SOCS pathways. Nature 548, 338–342. 10.1038/nature23450. 28792938 PMC5729908

[71] Bray NL, Pimentel H, Melsted P, and Pachter L (2016). Near-optimal probabilistic RNA-seq quantification. Nat. Biotechnol. 34, 525–527. 10.1038/nbt.3519. 27043002

[72] Miura M, and Chen H (2020). CUT&RUN detects distinct DNA footprints of RNA polymerase II near the transcription start sites. Chromosome Res. 28, 381–393. 10.1007/s10577-020-09643-0. 33070289 PMC7691310

[73] Morgens DW, Gulyas L, Mao X, Rivera-Madera A, Souza AS, and Glaunsinger BA (2025). Enhancers and genome conformation provide complex transcriptional control of a herpesviral gene. Mol. Syst. Biol. 21, 30–58. 10.1038/s44320-024-00075-0. 39562742 PMC11696879

[74] Liu N, Hargreaves VV, Zhu Q, Kurland JV, Hong J, Kim W, Sher F, Macias-Trevino C, Rogers JM, Kurita R, (2018). Direct Promoter Repression by BCL11A Controls the Fetal to Adult Hemoglobin Switch. Cell 173, 430–442.e17. 10.1016/j.cell.2018.03.016. 29606353 PMC5889339

