# Supplementary material for: Cytoplasmic mRNA decay by the antiviral nuclease RNase L promotes transcriptional repression
Source: Cell Rep. Author manuscript; Available in PMC 2026 Apr 20. (PMC13095223; doi:10.1016/j.celrep.2026.117028)
Supplement: 1 [file NIHMS2160117-supplement-1.pdf]

Cell Reports, Volume *45*

Supplemental information

Cytoplasmic mRNA decay by the antiviral nuclease RNase L promotes transcriptional repression

Xiaowen Mao, Sherzod Tokamov, Felix Pahmeier, Azra Lari, Jinyi Xu, Eva Harris, and Britt Glaunsinger

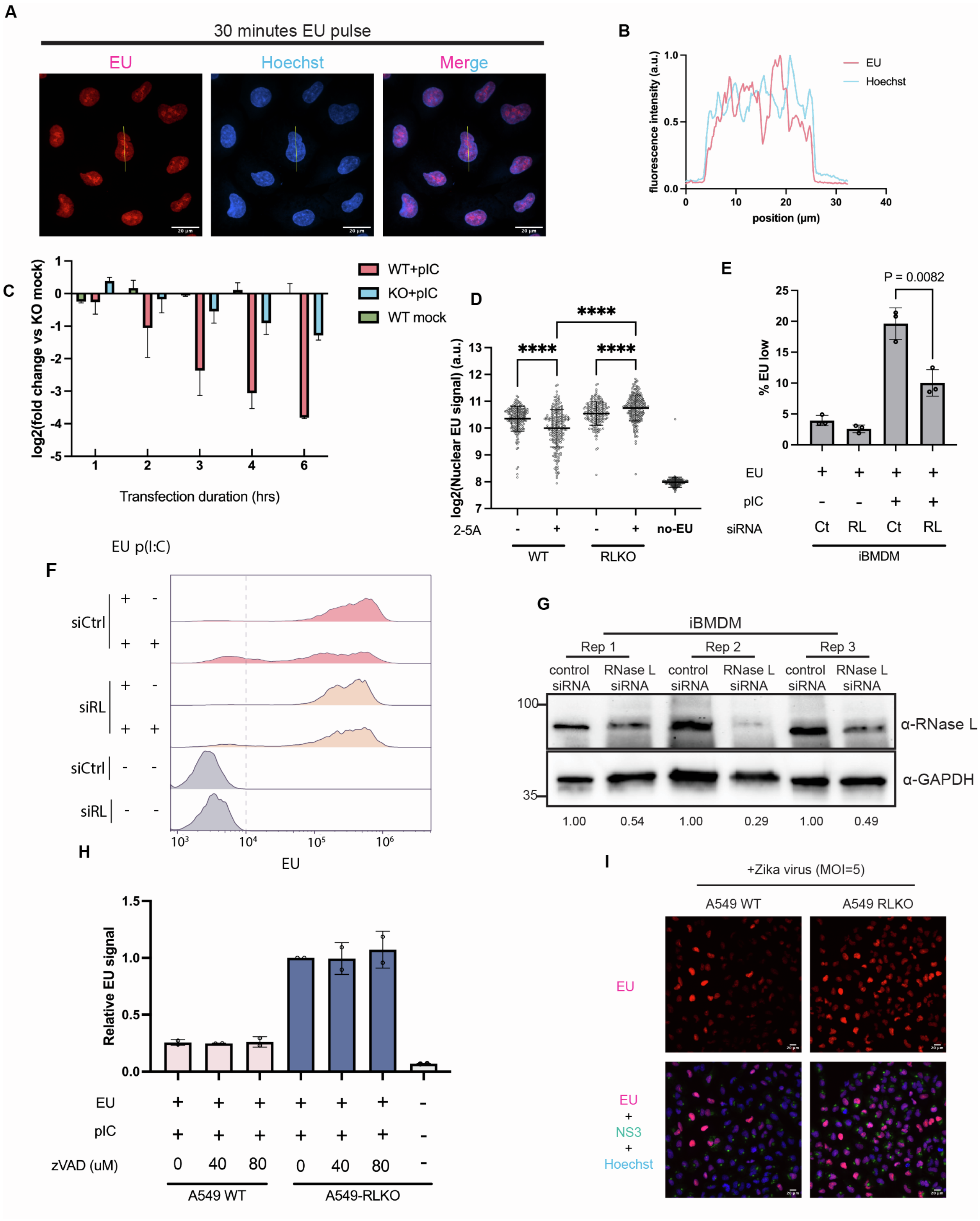

A
30 minutes EU pulse
EU
Hoechst
Merge
B
fluorescence intensity (a.u.)
position (μm)
EU
Hoechst
C
log2(fold change vs KO mock)
Transfection duration (hrs)
WT+pIC
KO+pIC
WT mock
D
log2(Nuclear EU signal) (a.u.)
2-5A
WT
RLKO
no-EU
E
% EU low
P = 0.0082
EU
pIC
siRNA
Ct
RL
iBMDM
F
EU p(I:C)
siCtrl
siRL
EU
G
iBMDM
Rep 1
Rep 2
Rep 3
control siRNA
RNase L siRNA
α-RNase L
α-GAPDH
1.00
0.54
1.00
0.29
1.00
0.49
H
Relative EU signal
EU
pIC
zVAD (uM)
A549 WT
A549-RLKO
I
+Zika virus (MOI=5)
A549 WT
A549 RLKO
EU
EU + NS3 + Hoechst

Figure S1. RNase L activation reduces nascent RNA progressively in A549 and iBMDM cells, independent of caspase activity. Related to Figure 1.

(A) Representative max-intensity-projected images of mock-transfected A549 WT cells pulsed with 1mM EU for 30 minutes. (B) Normalized fluorescence intensity across line trace in (*A*). (C) A549 WT or RLKO cells were either mock-transfected with RNAiMAX or transfected with 0.76µg/ml poly(I:C) for durations indicated, followed by a 30-min pulse labeling with 1 mM EU. Error bars represent the mean ± SD of log2-fold change over mock-transfected RNase L KO cells at the indicated time point. Data is from 2 biological replicates. (D) Quantification of nuclear EU signal in WT or RLKO cells, either mock-transfected or transfected with synthetic 2-5A. The data show one representative replicate of two independent biological replicates. Each dot represents the mean nuclear EU fluorescence intensity of one individual nucleus. Bars represent mean ± SD. ****, $p < 0.0001$; P-value was calculated using two-sided Welch's *t* test. (E) Quantification of the percentage of EU low cells. Error bars represent mean ± SD of 3 biological replicates. P-value was calculated using two-sided Welch's *t* test. (F) A representative flow cytometry histogram of murine iBMDM cells with treatments indicated. EU signal was gated according to no-EU controls, shown with a dashed line. (G) Western blot validation of RNase L siRNA knockdown efficiency. Numbers below lanes indicate relative levels of bands normalized to the control KD of the corresponding replicate. (H) A549 WT or RNase L KO cells were transfected with 0.76µg/ml poly(I:C) in the presence of z-VAD at the indicated concentration for 4 hours, followed by 30-min pulse labeling with 1mM EU. Each dot represents the median fluorescence intensity of the sample with indicated treatment from one biological replicate, normalized to the median fluorescence intensity of DMSO-treated KO cells. Bars represent mean ± SD. (I) Low magnification (20X) field of view of Zika virus-infected cells, from the same sample as *Figure 1D-E*.

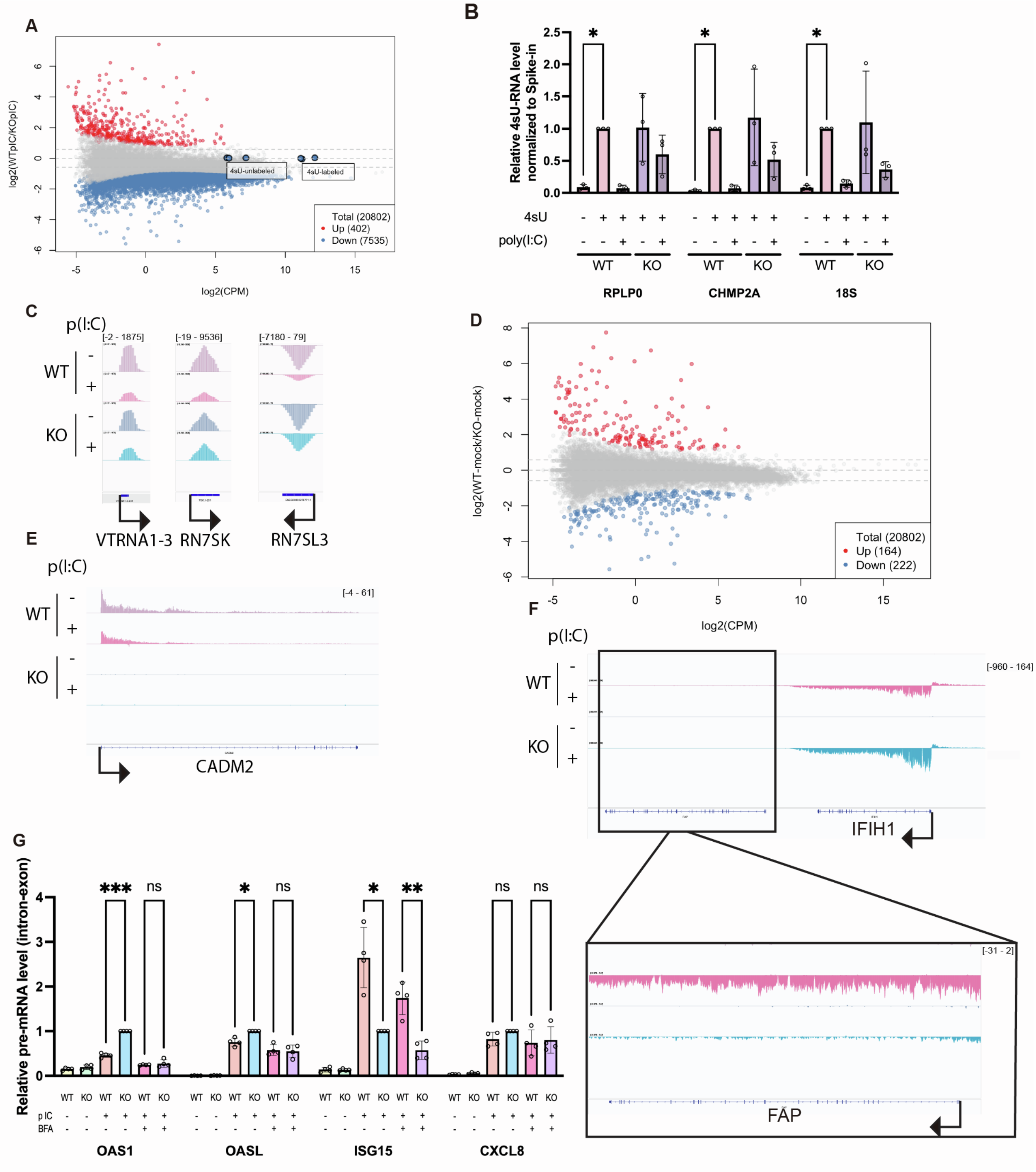
A
log2(WTpIC/KOpIC)
log2(CPM)
4sU-unlabeled
4sU-labeled
Total (20802)
Up (402)
Down (7535)
B
Relative 4sU-RNA level normalized to Spike-in
4sU
poly(I:C)
WT
KO
RPLP0
CHMP2A
18S
C
p(I:C)
[-2 - 1875]
[-19 - 9536]
[-7180 - 79]
VTRNA1-3
RN7SK
RN7SL3
D
log2(WT-mock/KO-mock)
log2(CPM)
Total (20802)
Up (164)
Down (222)
E
p(I:C)
[-4 - 61]
CADM2
F
p(I:C)
[-960 - 164]
IFIH1
[-31 - 2]
FAP
G
Relative pre-mRNA level (intron-exon)
p IC
BFA
ns
OAS1
OASL
ISG15
CXCL8

Figure S2. 4sU-seq data accurately captures changes on the nascent RNA level. Related to Figure 2.

(A) Differential expression analysis of 4sU-seq comparing WT cells to RLKO cells transfected with poly(I:C) cells (same as Figure 2D), with spike-ins highlighted. See Figure 2D for details. (B) RT-qPCR of 4sU samples compared to no-4sU control. RNA samples were the same as the ones used in 4sU-seq. *, $p < 0.05$ (ratio paired *t* test). (C) IGV tracks showing 4sU-seq signal coverage on RN7SK, VTRNA1-3 and RN7SL3, examples of downregulated Pol III transcribed genes. Tracks represent the average coverage of 3 independent biological replicates. In this and subsequent tracks, a positive value indicates coverage in the positive strand direction, while a negative value indicates coverage in the negative strand direction, and coverage depth is shown in brackets in the upper track. (D) Differential expression analysis of 4sU-seq comparing mock-transfected WT cells RLKO cells. (E) IGV tracks showing 4sU-seq signal coverage on CADM2, an example of cell line-specific expression variation. Tracks represent the average coverage of 3 independent biological replicates. (F) IGV tracks showing 4sU-seq signal coverage on FAP downstream of IFIH1, an example of read-through transcription. Tracks represent the average coverage of 3 independent biological replicates. (G) Pre-mRNA level of additional immune genes in WT or RLKO cells transfected with poly(I:C), with or without treatment of brefeldin A (BFA). Pre-mRNA level is determined by RT-qPCR using intron-exon spanning primer pairs, normalized to 18S. *, $p< 0.05$; **, $p<0.01$; ***, $p< 0.001$; ns, not significant; calculated with two-sided Welch's *t* test.

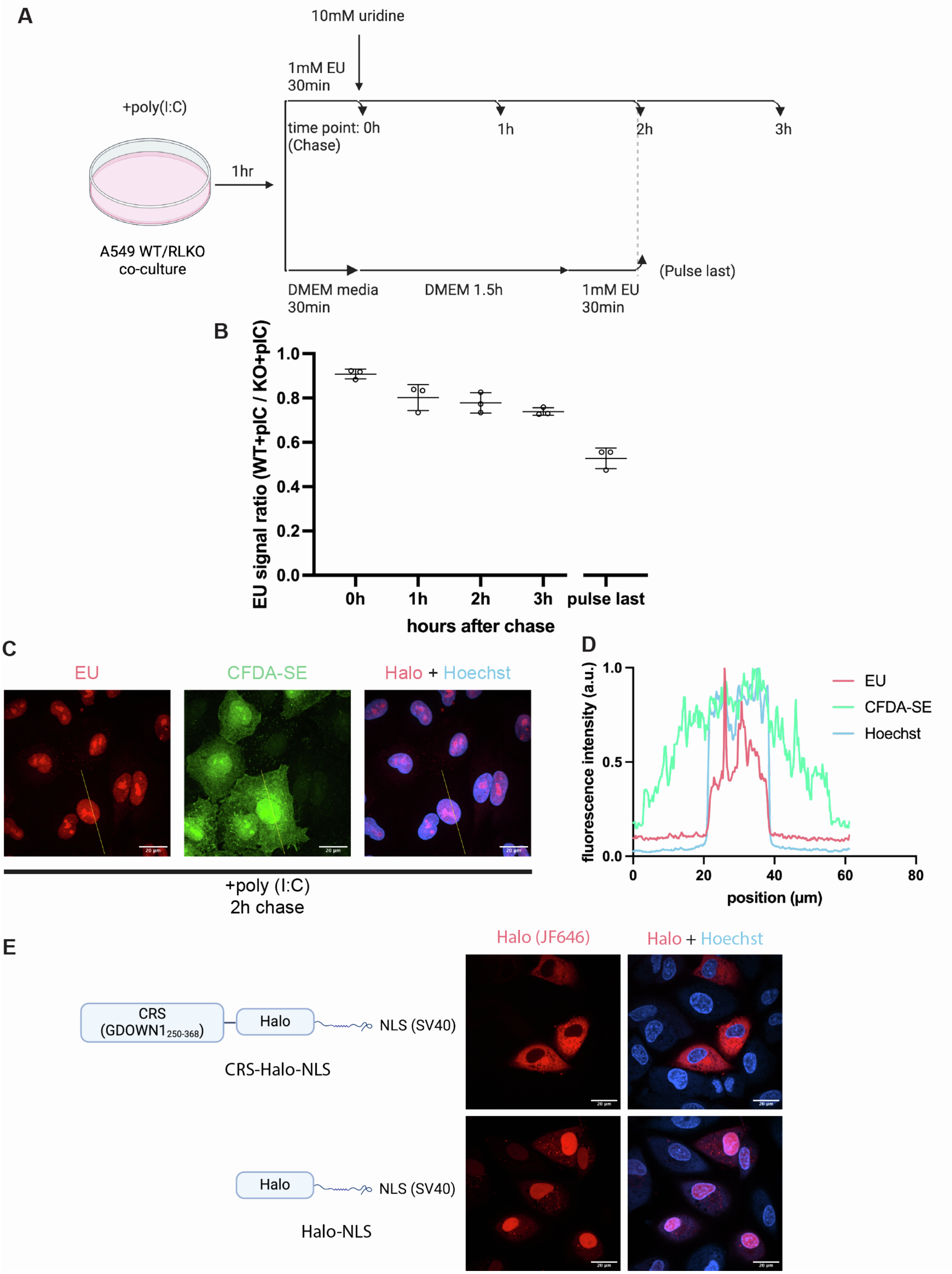

A
10mM uridine
1mM EU
30min
+poly(I:C)
time point: 0h
(Chase)
1h
2h
3h
1hr
A549 WT/RLKO
co-culture
(Pulse last)
DMEM media
30min
DMEM 1.5h
1mM EU
30min
B
EU signal ratio (WT+pIC / KO+pIC)
1.0
0.8
0.6
0.4
0.2
0.0
0h
1h
2h
3h
pulse last
hours after chase
C
EU
CFDA-SE
Halo + Hoechst
+poly (I:C)
2h chase
D
fluorescence intensity (a.u.)
1.0
0.5
0.0
EU
CFDA-SE
Hoechst
0
20
40
60
80
position (μm)
E
Halo (JF646)
Halo + Hoechst
CRS
(GDOWN1250-368)
Halo
NLS (SV40)
CRS-Halo-NLS
Halo
NLS (SV40)
Halo-NLS

Figure S3. EU pulse chase experiments accurately measure nuclear RNA turnover kinetics and functional confirmation of the CRS tag. Related to Figure 3.

(A) Diagram showing the experimental setup of the "EU pulse last" control in parallel to the EU pulse chase experiments shown in Figure 3A. (B) Quantification of EU signal in the "EU pulse at last" control. Data from Figure 3B, replotted as the ratio of EU signals in A549 WT to RLKO cells, are included for comparison. Each dot represents one biological replicate. Bars represent mean ± SD. (C) Representative max-intensity-projected images of cells transfected with poly(I:C) for 1 hour, pulsed with 1mM EU for 30 minutes then chased for 2 hours. WT cells were labeled with CFDA-SE dye and co-cultured with RLKO cells. (D) Normalized fluorescence intensity across line trace in *(C)*. CFDA-SE signal is included as a uniform distributed control for comparison. (E) Representative images of the localization of CRS-Halo-NLS and Halo-NLS. A single z plane is shown per construct. Diagram showing the constructs are included.

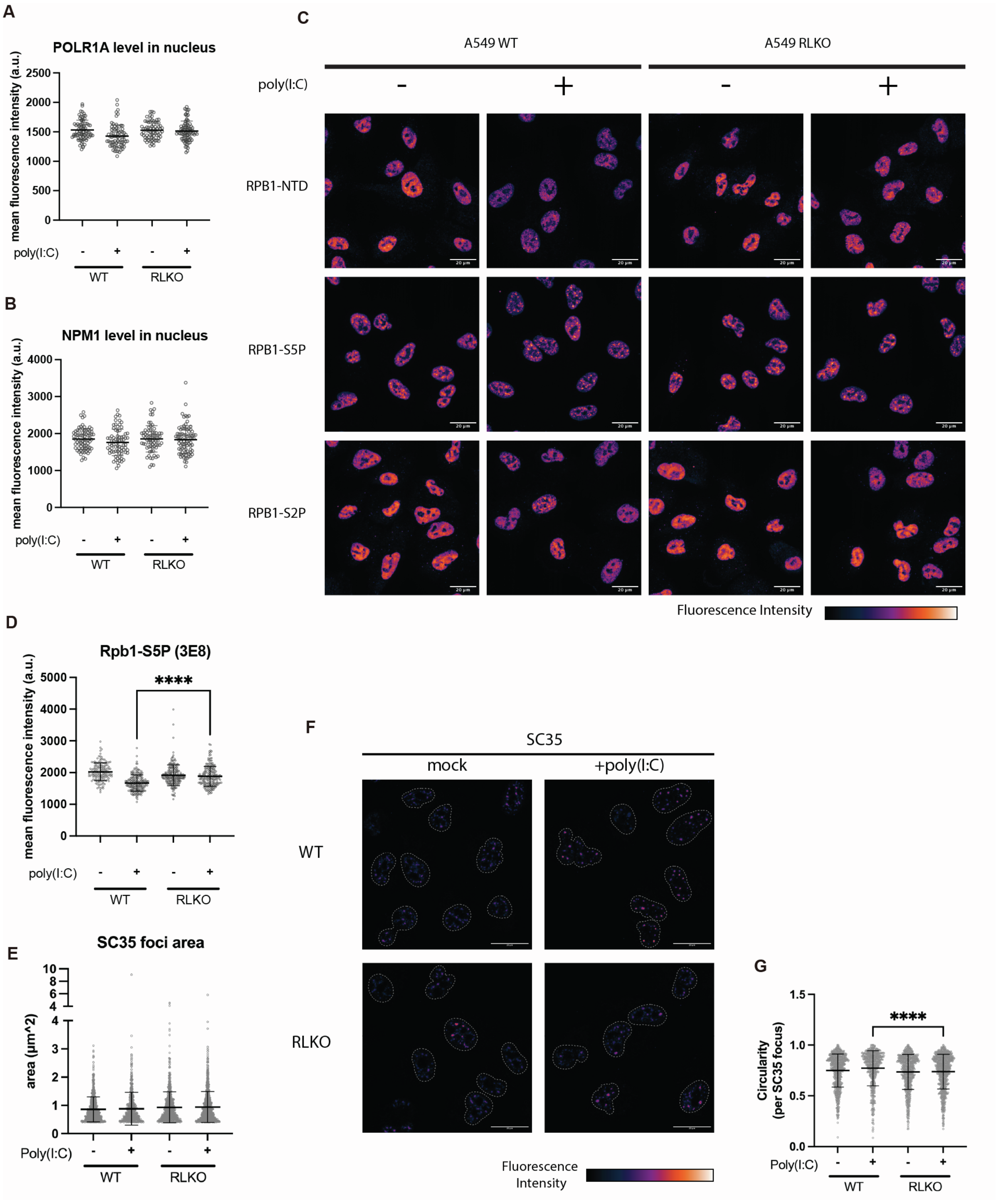
A
POLR1A level in nucleus
mean fluorescence intensity (a.u.)
poly(I:C)
WT
RLKO
B
NPM1 level in nucleus
mean fluorescence intensity (a.u.)
poly(I:C)
WT
RLKO
C
A549 WT
A549 RLKO
poly(I:C)
RPB1-NTD
RPB1-S5P
RPB1-S2P
Fluorescence Intensity
D
Rpb1-S5P (3E8)
mean fluorescence intensity (a.u.)
****
poly(I:C)
WT
RLKO
E
SC35 foci area
area (µm^2)
Poly(I:C)
WT
RLKO
F
SC35
mock
+poly(I:C)
WT
RLKO
Fluorescence Intensity
G
Circularity (per SC35 focus)
****
Poly(I:C)
WT
RLKO

Figure S4. RNase L activation reduces total and S5P Rpb1 levels in the nucleus and does not affect nuclear speckle size. Related to Figure 4.

(A-B) Quantification of mean POLR1A (A) and NPM1 (B) staining intensity in poly(I:C)-transfected A549 WT or RLKO cells. (C) Representative max-intensity-z-projection images of Pol II (NTD), S5P, and S2P staining in A549 WT or RLKO cells transfected as indicated. Data are the same as Figure 4D-F. (D) Quantification of mean S5P-RPB1 staining intensity using 3E8 antibody in poly(I:C)-transfected A549 WT or RLKO cells. Each point represents the mean intensity calculated for each nucleus. Error bars represent mean ± SD. ****, p<0.0001 (Welch's t-test). Data represents 1 replicate of 2 independent biological replicates. (E) Quantification of SC35 foci area for each SC35 focus using data in Figure 4D. Each point represents the value measured for each SC35 focus. Error bars represent mean ± SD. Data are from 1 representative replicate of 3 total replicates. (F) Max-intensity-z-projection images of SC35 localization in mock or poly(I:C)-transfected A549 WT or RLKO cells. Data from Figure 4G is included for comparison. (G) Quantification of mean SC35 circularity for each SC35 focus using data in Figure 4*G* and Figure S4F. Each point represents the value calculated for each SC35 focus. Error bars represent mean ± SD. ****, p<0.0001 (two-sided Welch's t-test).

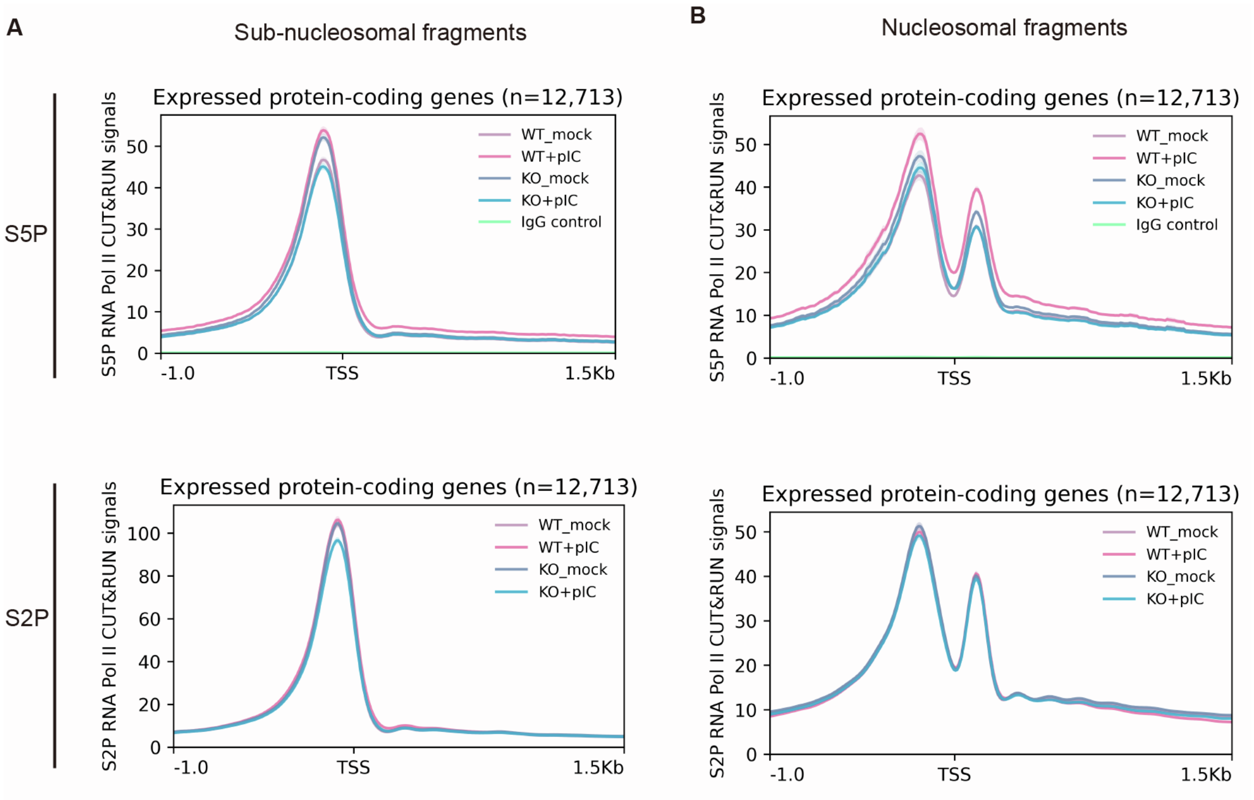

Figure S5. RNase L activation does not reduce CUT&RUN subnucleosomal and nucleosomal signals at TSS. Related to Figure 5.

(A) Metagene analysis of S5P and S2P Pol II CUT&RUN subnucleosomal fragment (<120bp) coverage at -1kb to +1.5kb around transcription start site (TSS) of expressed protein-coding genes. (B) Metagene analysis of S5P and S2P Pol II CUT&RUN nucleosomal fragment (>120bp) coverage at -1kb to +1.5kb around the transcription start site (TSS) of expressed protein-coding genes. The enrichment of subnucleosomal fragments slightly upstream of TSS and the bimodal peaks around the TSS of the nucleosomal fragments likely reflect the local nucleosome organization around Pol II occupancy sites.

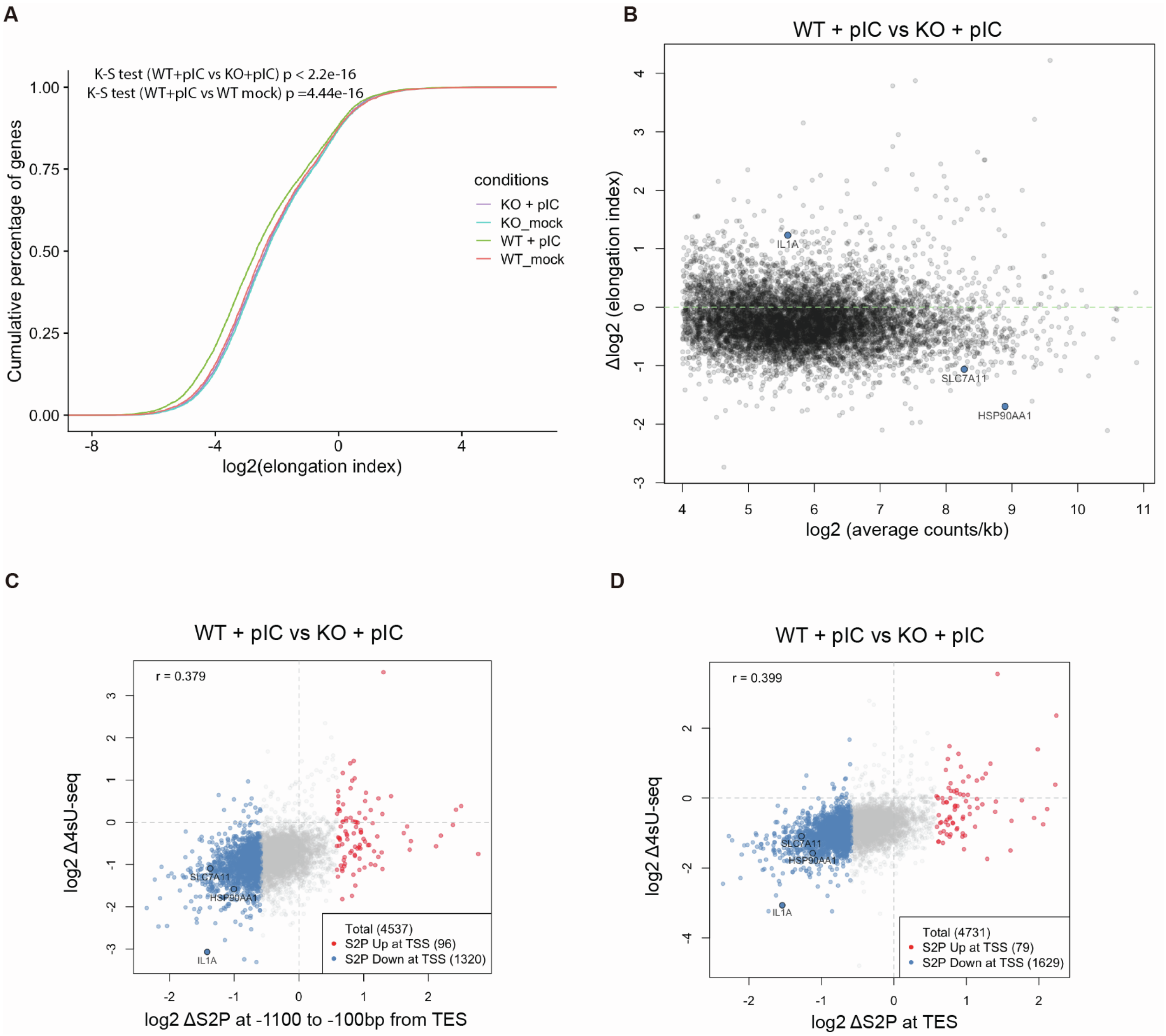

Figure S6. RNase L activation reduces S2P Pol II CUT&RUN signal in the gene body, which correlates with the reduction in 4sU-seq signal. Related to Figure 6.

(A) Empirical cumulative distribution function (ECDF) plot of log2(elongation factor) for WT or RLKO cells, either mock-transfected or transfected with poly(I:C). P values were calculated with two-sided Kolmogorov–Smirnov tests. (B) Scatterplot showing the changes in elongation index between poly(I:C)-transfected WT cells and mock-transfected WT cells in relation to the read density in the gene body. (C-D) Scatterplot showing the relation between log2 fold change of 4sU-seq and S2P Pol II occupancy at late (*C*) or TES windows (*D*). Up- or down-regulated S2P is defined in the same way as Figure 6F-G.
